# The impact of wage structure and ability on the incentives for practitioners to engage in primary care in China: a mathematical analysis based on the incentive mechanism of heterogeneous practitioners and tripartite evolutionary game

**DOI:** 10.3389/fpubh.2025.1527676

**Published:** 2025-06-18

**Authors:** Yuxun Zhou, Ren Long, Xiaoyun Liu

**Affiliations:** School of Public Health, Peking University, Beijing, China

**Keywords:** general practitioners, separating equilibrium, wage structure, performance-based wage, evolutionary stable points

## Abstract

**Objective:**

This study aims to analyze how wage structure and individual ability affect practitioners' participation in primary healthcare.

**Methodology:**

This study employs a mixed moral hazard and adverse selection model to analyze the optimal performance-based wage for general practitioners under separating and pooling equilibrium. Subsequently, we utilize tripartite evolutionary games to analyze the dynamic process of participation strategies to the primary health care with high—and less experienced practitioners.

**Findings:**

Our study yields four main findings: (1) Under an effective separating equilibrium, high-quality practitioners receive information rent, and there is no distortion at the top, while less experienced practitioners face allocation distortion. (2) When the performance risk of less experienced practitioners is greater than or equal to that of high-quality practitioners, reducing the performance risk of less experienced practitioners is an effective method of increasing their performance wage. Conversely, when the performance risk of less experienced practitioners is less than that of high-quality practitioners, and they can transform into high-quality practitioners by increasing education costs, they will be incentivized to continue as general practitioners, provided there is a precise promotion mechanism. (3) If reforms are made to the wage structure in primary healthcare, an effective approach is to increase the proportion of the floating part. This would lead primary healthcare institutions to choose contracts under separating equilibrium, encouraging high-quality practitioners to participate in primary healthcare and less experienced practitioners to improve their abilities by increasing education costs. However, the proportion of the floating part in the wage structure should not be excessively large. (4) The effective cost of medical resource utilization influences the wage structure, and establishing reasonable upper and lower limits for performance wages can effectively increase the incentive for high-quality practitioners to participate in primary healthcare.

**Significance:**

For the first time, our study employs a tripartite evolutionary game model to analyze the development of the general practitioner system. We analyze how the reform of the wage structure can encourage more practitioners to participate in primary healthcare. Our findings can lay the theoretical foundation for subsequent empirical analyses. Moreover, our findings provide theoretical assistance for government decisions and healthcare institutions.

## 1 Introduction

The health workforce crisis is a global issue. In Europe, this problem manifests as countries need help to retain existing healthcare personnel in their systems and face challenges in recruiting new healthcare workers. The health workforce crisis is driven by systemic issues such as low pay, poor working conditions, and widespread dissatisfaction among healthcare workers, as emphasized in reports by The King's Fund (1). These root causes have led to secondary effects including: (1) Aging workforce; (2) Increased absenteeism and resignations, particularly evident after the outbreak of COVID-19. The United Kingdom would need more than 571,000 healthcare professionals by 2036. Over the next 15 years, the U.K.'s National Health Service (NHS) is expected to see a reduction of 28,000 general practitioners (GPs), a decrease of 44,000 community nurses, and a more severe shortage of nursing staff (2). The United States faces a shortage of healthcare providers, particularly in primary care, dental, and mental health services (3). The U.S. Bureau of Labor Statistics predicts a shortage of 195,400 nurses by 2031, and by 2028, vacancies for home health aides and personal care aides will increase by 37% (4). A nursing workforce market study commissioned by the Australian government in 2021 predicts a shortage of over 200,000 full-time care workers in Australia by 2050 (5). The shortage of healthcare workers is increasingly becoming a pressing global issue that demands urgent attention.

Two reports from the World Health Organization (WHO) mention that economically underdeveloped regions, due to increased medical demands and the mass migration of healthcare professionals to economically developed regions, will experience a more significant health workforce crisis (6, 7). China also faces a slow development in family medicine, with financial incentives being the most significant factor influencing the choices of GPs (8–10). From a financial incentive perspective, From the perspective of financial incentives, Chinese healthcare workers primarily face three issues: (1) Migration of medical professionals to economically developed regions; (2) Migration of medical professionals to high-income healthcare institutions; (3) The issue of the wage structure of healthcare workers. Currently, in China, the wage structure of primary healthcare workers is characterized by a disproportionately large fixed portion and a tiny float portion. In contrast, the float portion of healthcare workers' wages in large hospitals is very significant, while the fixed portion is relatively small. The results in low work incentives for healthcare workers in primary healthcare settings. Conversely, in large hospitals, healthcare workers may waste healthcare resources in pursuit of performance incentives. Therefore, studying the impact of wage structures on primary healthcare development is a significant topic.

We provide a brief review of the current state of research on incentives for GPs, dividing it into two parts: a review of international literature in the first part and a review of China's literature in the second part.

The WHO's literature review report uncovered the development of theoretical research on physician incentives from 1989 to 2000. In this report, they reviewed 352 papers. They concluded that there was no evidence in these literature pieces from 1989 to 2000 that could serve as a basis for evaluating the impact of incentive interventions. Many studies needed a comprehensive description of incentive types and rarely provided reliable evaluation methods (11). Review studies during the same period, such as Town et al. (12), found that small rewards did not incentivize physicians. Verma et al. (13) conducted a systematic review of 184 eligible papers and studied the incentive effects of various financial incentive measures for GPs in different countries. They affirmed the effectiveness of financial incentive measures in some aspects but found insufficient evidence to support the incentive effects resulting from increased professional levels and support for medical education.

Ahmed et al. (14) studied the impact of the UK Quality Outcomes Framework (QOF) on GP incentives. They selected 42 papers from 22,087 studies for review and discussed the research topic from three directions: financial incentives, non-financial incentives, and competition. In terms of the impact of financial incentives, they analyzed the benefits and drawbacks of the QOF. The benefits to patients far outweighed the benefits to GPs. GPs generally expressed negative opinions about the QOF system, “*im-personalization of medicine, fragmentation of holistic care, weakened clinical leadership, and unfair distribution of funds*” [(14), p. 6] as factors leading to imbalances in healthcare distribution in the UK. Another significant reason is that due to a lack of resources, GPs in socioeconomically deprived areas find it challenging to meet QOF targets.

Furthermore, these practitioners receive less economic compensation in these areas, resulting in fewer resources available to improve the quality of care, creating a negative cycle. Heider and Mang (15) conducted a literature review of 21 related topics. They concluded that, based on these 21 literature reviews, there is no consistent evidence that financial incentive mechanisms clearly impact the quality of physicians' “production”. However, they believe that future focus should be on team incentives vs. individual incentives, cultural differences in different countries, and non-financial incentive factors.

China also faces financial incentive issues for GPs. Yip et al. (16) reviewed the development of China's healthcare reform over 10 years. They suggested that despite outstanding achievements in healthcare reform, gaps still exist in nursing quality, non-communicable disease control, service efficiency, healthcare expenditure control, and public satisfaction. One of their recommendations is to change the above gaps by adjusting the incentives and governance of hospitals and the primary healthcare system to improve the quality of primary healthcare providers. Jing and Fang (17) first mentioned the theoretical analysis of the incentive mechanism for Chinese GPs. Their MCGUIRE model analyzed the incentive issues faced by Chinese GPs and made recommendations. Yi et al. (18) conducted empirical analysis through Logistic regression on the signing of GPs in the Chuan-Qian region. They found that the promotion of the GP system could be faster, reflected in the low signing rate of GPs. They identified two reasons: (1) The low economic income of GPs results in a lack of incentive for GP signing; (2) Limited professional level and unclear career prospects for GPs. Feng et al. (19) conducted an empirical analysis of the incentive factors affecting GPs in different positions. They found that income and benefits are the most critical factors affecting GP incentives. Training opportunities and promotion conditions are the second most important factors.

In China, research on GPs also needs more theoretical analysis. Many studies use empirical analysis to analyze the willingness of GPs, and the impact of existing incentive mechanisms on GPs still needs to be explored. Jing and Fang (17) mentioned in their article, “*In incentive studies on Chinese family medicine, there is currently a lack of theoretical analysis on the behavior of GPs under different medical insurance payment methods for contracted services, and there is also a lack of research models constructing the incentive mechanisms affecting family doctor behavior*” [(17), p. 338].

The existing literature focuses on analyzing the factors that influence physician motivation. These factors come from both financial and non-financial incentives. However, more needs to be done to analyze the impact paths of these critical factors. Second, only some studies have comprehensively analyzed the driving force of doctors' participation in primary medical care from the perspective of both supply (GPs) and demand (primary healthcare institutions). The innovation of this study is as follows: (1) We analyzed the motivation of doctors to participate in primary care from the perspective of supply and demand. (2) We analyzed the influence path of critical factors influencing practitioners' motivation. (3) We take the wage structure as the primary analysis factor, which expands the financial incentive analysis theory of practitioners. Our theoretical analysis will fill the current gaps in the theoretical analysis of financial incentives for GPs, particularly regarding wage structures. We propose the following two research questions:

*RQ 1: How do wage structures and individual abilities affect GPs' performance?*
*RQ 2: How do different performance mechanisms influence GPs' participation in primary healthcare?*

Our study focuses on the participation level of GPs, financial incentives, and wage structure. The significance of our research lies in providing a theoretical basis for the theme of the shortage of GPs through theoretical analysis of the financial incentive issues they face. We can offer scientific theoretical support for future empirical analysis variables through theoretical analysis. Our research also has practical solid significance, as our findings will present a reliable incentive mechanism, aiding government and healthcare institutions in management and decision-making.

The structure of this paper is as follows: In the second section, we will first employ a mixed model incorporating moral hazard and adverse selection to analyze the optimal wage for practitioners under separating equilibrium. Second, we will use evolutionary game theory to construct a dynamic system of strategy choices for primary health institution and high-quality and less experienced practitioners. Third, we will analyze the dynamic changing process of practitioners' participation in China's PHC in the future through simulating the current situation in China and modifying the parameters. The Section 3 will discuss our mathematical results and provide practical significance and policy insights. Finally, we will summarize in the Section 4.

## 2 Methodology

### 2.1 Model establishing

To understand how financial incentives affect doctors' participation in primary healthcare, we constructed a two-part mathematical model. The first part uses economic theory to calculate what kind of salary structure would best motivate high- and low-skilled general practitioners (GPs), taking into account factors like risk, effort, and performance. The second part uses evolutionary game theory to simulate how doctors and health institutions make decisions over time. For example, we look at how high- and less experienced practitioners respond to different pay systems, and whether institutions prefer contracts that reward good performance (called separating equilibrium) or treat all doctors the same (pooling equilibrium). By comparing these strategies, we can predict how policy reforms—like adjusting the balance between fixed salary and performance pay—might influence who chooses to work in primary care. The full mathematical derivations are provided in the Appendix for interested readers.

The fixed-to-floating wage structure, the classification of healthcare personnel, and the specification of effort-related costs are designed in accordance with the realities of China to reflect the institutional characteristics of its primary healthcare system. These include a predominantly public-sector employment model, limited flexibility in salary adjustments, and a performance evaluation mechanism guided by administrative benchmarks. Accordingly, the model is specifically developed to capture the unique behavioral patterns and strategic dynamics of China's healthcare labor market.

### 2.2 Numerical analysis

We categorize θ into three levels based on specialty degree or below, bachelor's degree, and postgraduate degree or above (20): (0, 0.33] is the level 3 (specialty degree or below); (0, 33, 0.65] is the level 2 (bachelor's degree); (0.65, 1) is the level 1 (postgraduate degree or above). In our earlier assumptions, practitioners are risk-averse (ρ>1). According to corollary 2, we need to ensure θ_*L*_*B*_*L*_σ_*H*_ = θ_*H*_*B*_*H*_σ_*L*_ during the parameterization process. To ensure fairness in performance, the performance coefficients of heterogeneous practitioners are the same within the same institution. Once θ_*L*_ and θ_*H*_ are given (based on the fact that there is a significant educational gap between practitioners in grassroots medical institutions and tertiary hospitals, we assume θ_*H*_ = 0.85, θ_*L*_ = 0.25), we can determine the values of σ_*H*_ and σ_*L*_. Simultaneously, according to corollary 2, we need to ensure that $c\in \left(0,\;\frac{\theta_{H}^{2}B_{H}^{2}}{\rho \sigma_{H}^{2}}\right]⋂\left(0,\;\frac{\theta_{L}^{2}B_{L}^{2}}{\rho \sigma_{L}^{2}}\right]$. Keeping other parameters fixed, assuming a significant difference in Δθ (when θ_*L*_∈(0, 0.33]), we take *m* = 0.05, 0.2656, 0.75, respectively, where *m* = 0.2656 is the optimal result calculated through corollary 1. In other words, we have calculated the optimal wage structure, *m* = 0.2656, based on parameters derived from the actual situation in China. Table 1 shows the value of parameters' setting.

**Table 1:** Value of each parameter.

**Parameters**	**Value**	**Sources**
*P*	0.037	(20 )
β	0.35	Assumption
θ_*H*_	0.85	Assumption
θ_*L*_	0.25	Assumption
*m*	0.05/0.2656/0.75	Assumption and calculate according to the corollary 1
*w*_0, *H*_	0.3295	(20)
*w*_0, *L*_	0.3295	(20)
*B*_*H*_	1.4	Calculate according to the corollary 1
*B*_*L*_	1/0.3678	Calculate according to the corollary 1
*c*	0.12/0.08	Calculate according to the corollary 1
ρ	1.5	Assumption
$\sigma_{H}^{2}$	2.06	(48)
$\sigma_{L}^{2}$	1.84	(48)

**Figure 1:**
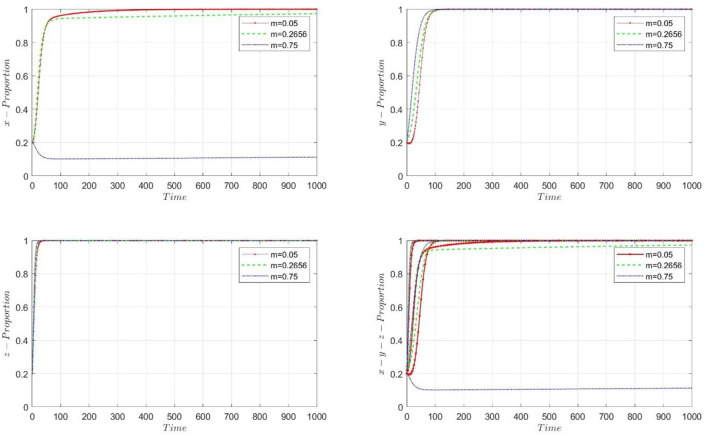
The dynamic progress of proportion, θ_*L*_ = 0.25, and *m* is in different value.

Figure 1 illustrates the dynamic selection process of high-quality practitioners, Less experienced practitioners, and PHC institutions as *m* takes different values. We assume PHC institutions select the performance mechanism associated with high-quality practitioners. We observe that when *m* = 0.05 and 0.2656 (i.e., a higher proportion of the floating part in the wage structure), the selection probabilities of all three parties converge rapidly to 1. It implies that with a higher proportion of the floating part, PHC institutions will eventually choose the contract mechanism under separating equilibrium, and less experienced practitioners will opt to work in PHC institutions. The selection of high-quality practitioners fluctuates, but ultimately, they still choose to accept employment in PHC institutions. As the proportion of the floating part in the wage structure gradually increases (*m* increases), the probability of PHC institutions choosing the performance mechanism under separating equilibrium decreases. When *m* = 0.75, PHC institutions choose the performance mechanism under pooling equilibrium. Regardless of the wage structure, less experienced practitioners will choose to work in PHC institutions.

**Figure 2:**
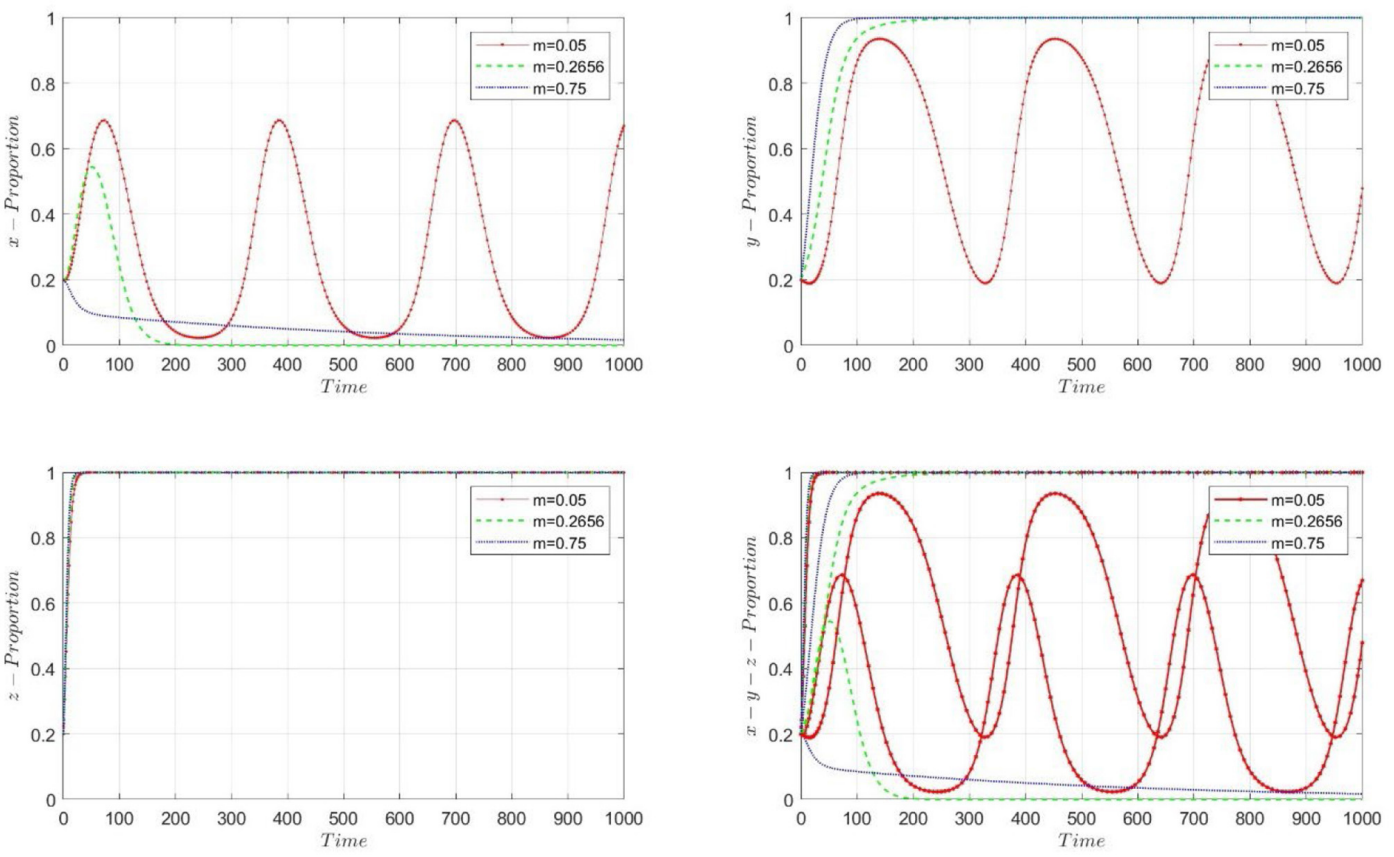
The dynamic progress of proportion, *m* is in different value, when the PHC institutions choose to pooling to the less experienced practitioners' performance wage mechanism.

PHC institutions choose to merge into the performance mechanism associated with less experienced practitioners, and we consider *m* = 0.05, 0.2656, and 0.75, respectively. Figure 2 illustrates the convergence of selection probabilities for the three participants as m takes different values. When *m* = 0.05, the selection probabilities for PHC institutions and high-quality practitioners do not converge. PHC institutions choose the performance mechanism under pooling equilibrium when *m* = 0.2656 and 0.75. It aligns with the theoretical results in Section 2.1. In corollary 3, we obtain a result that only when PHC institutions merge into the performance mechanism associated with high-quality practitioners (1, 1, 1) is an ESS point.

**Figure 3:**
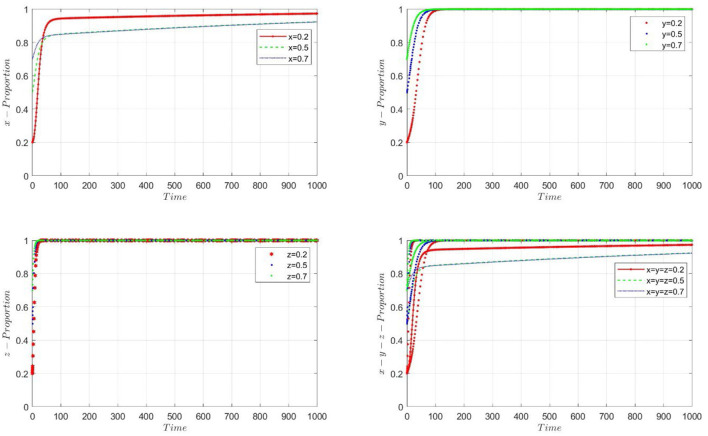
The stability test when given the different initial value for *x, y, z*.

**Figure 4:**
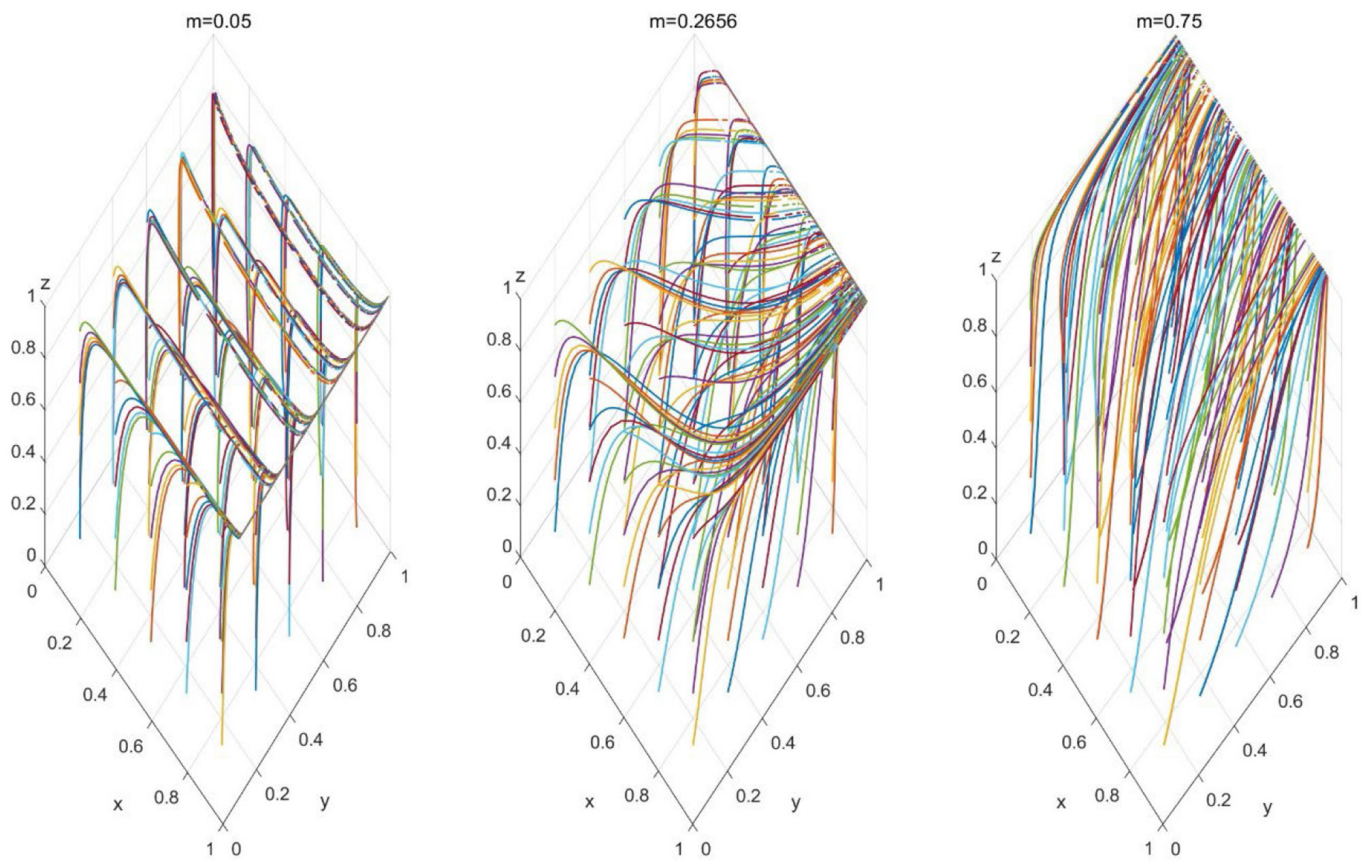
Three-dimensional evolutionary strategies, when *m* = 0.25, 0.2656, 0.75.

We examined the stability of the model by selecting different initial values. Figure 3 demonstrates the impact of initial value changes on the probability changes when *m* = 0.2656. By setting *x*, *y*, and *z* to 0.2, 0.5, and 0.7, respectively, we found that although the convergence speeds of *x*, *y*, and *z* varied under different initial values, the selection probabilities of the participants eventually converged to 1. Thus, the convergence results of the model are not affected by changes in the initial values. Figure 4 illustrates the three-dimensional strategy's evolution when PHC institutions merge into the performance mechanism associated with high-quality practitioners, with *m* = 0.05, 0.2656, and 0.75.

China's current GPs' wage structure comprises a large proportion of fixed wages and a small proportion of floating wages, i.e., θ_*L*_ = 0.25, *m* = 0.75 (a large proportion of fixed part in the wage structure). To further promote the development of PHC, we aim to encourage more high-quality practitioners to participate. Therefore, based on the dynamic system simulation of our evolutionary game; to achieve the equilibrium (1, 1, 1), we need to reduce the proportion of the fixed part in the PHC's wage structure. When the proportion of the fixed part decreases, on the one hand, since the only choice for less experienced practitioners is to continue serving in PHC, reducing the proportion of the fixed part will prompt them to increase their education costs. On the other hand, due to their higher ability, high-quality practitioners will be incentivized to work in PHC with an increase in the proportion of the floating part in the PHC's wage structure (more work, more pay). According to simulations of our tripartite evolutionary model, a crucial method to encourage more high-quality practitioners to work in PHC is to reform the PHC's wage structure by increasing the proportion of the floating part. Such reforms enhance the incentives for more high-quality practitioners to work in PHC and improve the ability of less experienced practitioners.

## 3 Implications and discussions

While the model provides valuable insights into incentive structures in China, caution must be exercised in generalizing its findings to other national contexts. For instance, in the UK, the National Health Service (NHS) offers fully public healthcare with standardized salaries and career pathways, whereas in the US, healthcare delivery is largely dominated by private-sector providers operating under market-based compensation. These systemic differences may affect how practitioners respond to financial incentives, information asymmetries, and contractual structures. Therefore, adaptation of the model to other settings would require structural modifications and empirical recalibration.

Our propositions 1 and 2 address our research questions 1 and 2. Proposition 1 answers the question of what factors determine the performance of heterogeneous GPs (RQ 1). In proposition 1, we discovered that one effective way for less experienced practitioners to increase their performance wage under the separating equilibrium is to increase their educational costs (narrowing Δθ). When there is a clear incentive mechanism to distinguish between practitioners' types, the performance wage of high-quality practitioners is different from that of less experienced practitioners. Less experienced practitioners can achieve the same performance wage as high-quality practitioners by increasing their educational costs, i.e., θ_*L*_ = θ_*H*_, when the performance risk of high-quality practitioners equals that of less experienced practitioners, namely, $\sigma_{H}^{2}=\sigma_{L}^{2}$. Suppose $\sigma_{L}^{2}<\sigma_{H}^{2}$, we have $w_{H}\left(t\right)=\frac{\theta_{H}\left(1-m\right)B_{H}\beta }{\theta_{H}^{2}{\left(1-m\right)}^{2}B_{H}^{2}+c\rho \sigma_{H}^{2}}<w_{L}\left(t\right)=\frac{\theta_{H}\left(1-m\right)B_{H}\beta }{\theta_{H}^{2}{\left(1-m\right)}^{2}B_{H}^{2}+c\rho \sigma_{L}^{2}}$. The situation implies that additional high-quality practitioners will choose to work in PHC institutions because, under this incentive mechanism, the exact education costs will yield more performance rewards in PHC institutions (due to lower performance risk). When $\sigma_{L}^{2}>\sigma_{H}^{2}$, a less experienced practitioner, due to the higher performance risk, may still lack the incentive to choose to enhance their ability. Only when the increase in benefits from less experienced practitioners improving their ability exceeds the decrease in income caused by the risk will they choose to enhance their ability (i.e., increase educational costs). The finding is contingent upon PHC institutions adopting the separating equilibrium performance mechanism (i.e., a higher proportion of the floating part in the wage structure).

Many developed countries have implemented unified doctor training programs. The success of these programs supports our theoretical findings. The Singapore health system emphasizes continuing medical education (CME), requiring practitioners to attend regular training and refresher courses. Singapore's Ministry of Health works with several universities and medical institutions to provide practitioners with extensive training resources and funding. Through these trainings, practitioners can update their medical knowledge and techniques to improve the quality and efficiency of care. For example, Leong et al. (21) studied the effect of practitioners on the clinical quality of radiation oncology after receiving CME. They found that the clinical quality of radiation oncology treatment was significantly improved after receiving CME. In the United States, Residency Programs are an essential way to upgrade the skills and knowledge of practitioners. These training programs are usually run in collaboration between hospitals and medical schools and cover various medical fields. Residents undergo rigorous assessment and evaluation in the training process, and medical standards are improved through continuous learning and practice. Some studies (14, 22, 23) have shown that well-trained GPs receive significantly higher merit wages after completing their training than GPs who do not receive the same training. Batalden and Davidoff (24) researched quality control in the medical field. Their conclusions are consistent with the findings of this paper. They believe that through continuous education and training, the quality of medical care can be continuously improved. After receiving education and training, practitioners can master the latest medical knowledge and technology and provide higher medical services, thus increasing their performance wages.

We discuss and compare our findings with existing research. Worldwide, there is a prevalent health workforce crisis. Low wage is one of the primary and critical factors contributing to the crisis. In the United Kingdom, only 25% of NHS staff reported being satisfied with their pay. Insufficient wages lead healthcare workers to feel undervalued. According to a report by the U.S. Centers for Disease Control and Prevention, nearly half of healthcare workers reported experiencing burnout in 2022, an increase compared to 2018 (25, 26). The income disparity has led to the migration of healthcare workers from low-income regions to more affluent areas. Agyeman-Manu et al. (27) mentioned that increasing health workers are migrating to high-income countries, leading to a shortage of health workers in low-income and underdeveloped areas.

In China, health workers face the choice of working in tertiary hospitals or PHC institutions, economically developed or underdeveloped regions. It still boils down to the issue of financial incentives for health workers (one of the crucial reasons). The health worker incentive problem in China can be described as follows: health workers prefer to choose higher-paying and better-conditioned tertiary hospitals or economically developed regions. As described by Yuan et al. (28): “*Physicians working in hospitals, particularly at the tertiary level, have higher social respect, receive more opportunities for career development and are better paid*” [(28), p. 8]. It leads to a scarcity of healthcare workers in the primary healthcare system, particularly in economically underdeveloped regions (the scarcity here implies two aspects: (1) scarcity of healthcare workforce; (2) scarcity of high-quality healthcare workforce). Patients are more inclined to seek medical treatment in economically developed regions or well-equipped, high-standard tertiary hospitals. It results in a lack of consumers for primary healthcare institutions, further causing a decrease in the performance income of primary care practitioners. Therefore, primary care practitioners lack incentives to choose primary healthcare institutions. The entire incentive system forms a vicious cycle. Through separating equilibrium, we designed an incentive mechanism. Proposition 1 establishes a feasible separating equilibrium that allows the identification of doctor categories and ensures that healthcare workers of various categories have no incentive to disguise themselves as other categories. As mentioned earlier, the core of the incentive mechanism is that different levels of professional competence among practitioners' lead to different performance wages. When less experienced and high-quality practitioners have the same risk level, enhancing their professional competence is critical to increasing their performance wages. Many existing research findings support our theoretical findings. Russell et al. (29) reviewed 34 papers out of 2,649 in their analysis. They found that professional training can increase the retention rate of healthcare personnel in rural areas, and training rural students and providing training in rural locations can also increase their likelihood of staying in rural areas for medical practice. Brown and McIntyre (30), through their study of the Australian PHC Research, Evaluation, and Development (PHCRED), discovered that encouraging community practitioners to conduct research can improve community healthcare quality and increase community practitioners' retention rate. They argue that enhancing the research level and motivation of GPs contribute to elevating the status of GPs, addressing the perception that “*general practice is one of the most intellectually underdeveloped disciplines in medicine*” and countering the notion that it is “*a lost cause*” [(31), p. 169]. They believe that improving the research skills and motivation of GPs is essential for raising the profile of this medical discipline.

Another meaningful way for less experienced practitioners to increase performance wages in situations with different risks is to reduce performance risk. According to our assumptions in Section 3, risk is assumed to be the variance of wages. For practitioners, risk may stem from uncertainty in patient sources. For example, general practitioners may not secure enough signed contracts, and thus, having a stable contract growth rate is a critical way to reduce the performance risk for GPs. Healthcare workers face the risk of failing to consistently deliver high-quality services due to factors such as fatigue, insufficient skills, and emotional stress. This represents the potential for performance fluctuations or decline resulting from environmental pressures, limited capabilities, and inadequate support. The National Health Service (NHS) in England has taken several measures to reduce the performance risk of less experienced practitioners. First, the NHS has tried to improve the working environment for practitioners. The NHS has sought to improve healthcare workers' sleep quality by adjusting shift schedules. Research indicates that limiting shift duration to 16 h can increase sleep time and reduce fatigue, thereby contributing to better overall health and job performance among medical professionals (32). The NHS has also implemented the CLEAR program to provide staff with greater opportunities for career development, potentially enhancing their wellbeing and thereby increasing job resources. Changes in job resources are among the key factors influencing performance risk (33). Another typical example is Kaiser Permanente's performance improvement system in the United States. They reduce the practitioners' workload through teamwork and technical support. They also provide a variety of training and support programs to improve the overall capacity of practitioners, thereby reducing performance disparities. Finally, they enhance team collaboration and introduce assistive technologies to reduce the effort and time required for practitioners to complete tasks, reducing overall performance risk. These cases prove that reducing practitioners' performance risk can improve their performance.

However, this involves some other issues. To obtain enough signed contracts, GPs must have the following characteristics: (1) Patients have a sufficient understanding of the service forms and content provided by GPs; (2) Patients have sufficient trust in the professional competence of GPs; (3) Patients themselves have sufficient financial capacity to sign contracts. These issues remain unresolved. These three topics are expected to be highly promising in future research. Through Proposition 2, we obtain two policy insights: 1. The most essential way to enhance the incentive for GPs is to improve their professional competence. Our conclusion aligns with the efficiency wage theory. High wages will attract high-quality workers (34). The heterogeneous labor model proposed by Weiss (35) suggests an increasing relationship between worker ability and the wages they receive. Since companies cannot distinguish workers' abilities, they may choose not to lower wages to retain workers, leading to a pooling equilibrium with adverse selection.

Our Proposition 2 directly concludes that the individual ability of practitioners will impact their performance wages and answers our RQ 2. We find that wage structure plays a crucial role in influencing GP participation in PHC. Generally, a high proportion of the floating part can promote greater GP participation in PHC. We analyzed our designed performance mechanism through a simulation based on China. However, we do not believe that a higher proportion of the floating part is always better. When this proportion exceeds a certain threshold, the incentive for GPs diminishes. This is due to healthcare costs: when GPs aim for higher wages through high performance, the associated medical costs are also transferred to them. This outcome is determined by the mechanism design. Therefore, our performance mechanism can limit GPs from wasting medical resources in pursuit of high performance. Additionally, according to our performance mechanism, a GP's ability is another major factor influencing their participation in PHC. When PHC implements the performance mechanism under a separating equilibrium, less experienced GPs will improve their abilities to adapt to the mechanism; otherwise, they will face net education costs.

However, according to the efficiency wage theory, other factors may also affect practitioners' efficiency wages. A crucial explanation is that practitioners may not be unable to obtain sufficient performance wages due to inadequate professional competence. Instead, practitioners may possess high professional competence; however, due to the unfairness of incentive mechanisms, practitioners withdraw effort. In other words, practitioners believe that the existing incentive wages only require a certain level of effort, and they choose to withdraw some remaining effort when comparing their wages with those of other peers. It leads to a situation where some GPs slack off or adopt a “laid-back” attitude. This occurrence is not due to their lack of ability but is a result of the unfairness of the incentive system (36). A significant source of this unfairness is the imbalance in wage structures, which can be categorized into fixed and floating parts. If the fixed part dominates, practitioners may lack the incentive for them to make an effort. Even if practitioners make efforts, they can only receive a fixed wage under this wage structure. This phenomenon is widespread in China's primary healthcare system. China has already begun implementing a signaling mechanism in the medical industry to screen high-quality and less experienced practitioners. The principle of fairness implies that even with the implementation of a signaling mechanism, the unfairness of the incentive mechanism may still cause practitioners to choose to “live simply”. It is a crucial topic to focus on while promoting the signaling mechanism.

To prevent performance-based pay structures from causing burnout or dissatisfaction among GPs, particularly in resource-limited rural areas, it is crucial to balance financial incentives with institutional support. Adjusting performance metrics to reflect local healthcare realities—such as incorporating qualitative indicators alongside quantitative targets—can reduce excessive pressure while ensuring service quality. Additionally, setting realistic performance thresholds, providing career development opportunities, and maintaining a hybrid wage structure that blends fixed salaries with performance incentives can enhance job stability and motivation. Strengthening administrative and peer support systems further alleviates workload stress, fostering a more sustainable and resilient PHC workforce.

While financial incentives can effectively stimulate individual behavior in the short term, they exhibit notable limitations. A key concern is the “crowding-out effect”, wherein monetary rewards may diminish intrinsic motivation, particularly in tasks driven by altruism or a sense of purpose (37). For instance, one study found that low monetary incentives led to worse performance in prosocial tasks compared to no incentives at all. Moreover, although financial incentives may increase participation in specific contexts—such as COVID-19 vaccination—their long-term impact is not significantly superior to non-financial interventions (38, 39). The effectiveness of financial incentives also varies across income groups: in the recruitment of physicians, the impact of financial rewards differed substantially based on the income level of the target population, indicating that financial incentives are not universally effective (40).

In contrast, non-financial incentives often play a more effective role in sustaining motivation and promoting behavioral change over time. Factors such as a supportive work environment, recognition of task significance, and a manageable workload can significantly enhance individual engagement and performance (37, 41). Furthermore, leadership style can substantially influence the effectiveness of incentives. Transformational leadership, in particular, fosters team collaboration and facilitates knowledge sharing, highlighting the importance of organizational culture and managerial practices in shaping long-term motivational outcomes (42).

Policies should focus on addressing the underlying systemic challenges within primary healthcare to create a more sustainable and supportive working environment for GPs. Reducing excessive administrative tasks is another crucial aspect to consider. Many GPs face a significant non-clinical workload, including paperwork, compliance documentation, and bureaucratic procedures, which negatively impact healthcare quality. The administrative burden may prevent GPs from focusing on patient care and maintaining high medical standards. Despite concerns about their short-term drawbacks, digital health technologies, automated data systems, and task delegation remain promising tools for improving efficiency in healthcare delivery. Evidence suggests that their implementation can initially lead to increased system interoperability issues, resistance to change, and cognitive load and workflow disruption, particularly when integration is poor or technical support is lacking (43–45). Nevertheless, when carefully designed and supported with adequate training and infrastructure, these technologies have the potential to reduce administrative workloads and enable general practitioners to devote more time to clinical care and patient interactions (43).

Ensuring continuous professional development opportunities is essential for maintaining high-quality primary healthcare. Policies should promote structured career pathways, professional training programs, and access to medical research and innovation. Establishing mentorship programs, academic collaborations, and government-subsidized training initiatives can enhance GPs' expertise while improving job satisfaction and retention rates.

After obtaining the separating equilibrium result from Proposition 1, we use evolutionary game theory to derive Proposition 2. Proposition 2 is another core finding of this paper, addressing the strategic choices of high-quality and less experienced practitioners under different wage structures. We obtain two evolutionarily stable strategies (ESS) with practical significance: (1, 1, 1) and (0, 0, 1). When $\pi_{JJ}^{PE}<\pi_{JJ}^{SE}$ is satisfied, PHC institutions will choose to implement the performance mechanism under separating equilibrium, and both high-quality and less experienced practitioners will choose to participate in primary healthcare. When $\{\begin{array}{c} \pi_{RJ}^{SE}<\pi_{JJ}^{PE}\\ \pi_{j}^{SE}<0 \end{array}$ is satisfied, PHC institutions will close the labor market for high-quality practitioners and only sign contracts with less experienced practitioners. Our goal is to study the impact of changes in the wage structure (*m*) on the incentive for participants to enter, and due to the complexity of analytical solutions, we use numerical solutions to study *m*.

The game eventually converges to (0, 0, 1) when the fixed part dominates. PHC institutions will close the market for high-quality practitioners' labor (not as an active closure but as a passive closure resulting from a high proportion of the fixed part, making high-quality practitioners unwilling to participate). In this equilibrium, only less experienced practitioners exist in primary healthcare; less experienced practitioners in this wage structure have no incentive to make efforts. Regardless of their efforts, they can only receive the fixed wage due to the wage structure with a high fixed part proportion. To develop the primary healthcare system, we hope to have more high-quality practitioners participating. Therefore, our goal is for the game to converge to (1, 1, 1). Through numerical analysis, we find that when the floating part dominates, primary healthcare institutions will adopt the performance mechanism under the separating equilibrium if practitioners choose to enter primary healthcare. They need to accept the “more effort, more gain” rule. For high-quality practitioners, a high proportion of the floating part can increase their incentive to enter primary healthcare because of their higher ability. The “more effort, more gain” rule will increase their performance income. For less experienced practitioners, many floating parts and minimal fixed part income mean they cannot “sit and wait”. However, as less experienced practitioners, the “more effort, more gain” rule implies that they must have more patients and provide better services. Therefore, after PHC institutions implement the performance mechanism under the separating equilibrium, less experienced practitioners will reduce the ability gap with high-quality practitioners through further learning, thus gaining more patients.

Corollaries 2 and 3 further elucidate the performance mechanism of heterogeneous practitioners under the separating equilibrium. Lemma 2, through the connection between wage structure and optimal wage, explains that there is no monotonic relationship between wage structure and performance wage. Instead, they exhibit a “parabolic” relationship. It indicates that “the greater the effort, the greater the performance” is not necessarily true. As described in the second section, the main reason for this phenomenon is that effort cost follows a quadratic cost function. This nonlinear cost function produces a nonlinear relationship between effort cost and wage performance. Incentives for GPs to exert effort led them to increase effort for higher performance. Increased effort leads to an increase in effort cost. According to our previous assumption, effort cost includes medical resource waste (ineffective medical resource utilization) caused by performance. It explains why setting a too-low proportion of the floating part is not feasible, and it also elucidates what kind of wage structure can optimize performance wage. Corollary 3 provides a theoretical basis for explaining the issue of upper and lower limits of wages in another context in China. We have designed a reasonable wage ceiling and floor through the separating equilibrium and optimal wage structure, which can maximize the incentives for GPs within this range. Corollary 2 also implies that once the outcome of tripartite participation is formed, the dynamic system will adopt the optimal wage structure to maximize practitioners' performance wage. Through numerical analysis, we find that the optimal proportion of the fixed part is ~0.2656.

In Canada, many practitioners are paid on a fee-for-service system. It is a high-percentage floating section wage structure. The system incentivizes practitioners to prescribe more drugs, perform more tests and surgeries, and sometimes perform unnecessary medical procedures to boost their income. For example, Allin et al. (46) showed a significant relationship between the high C-section rate in Canada and practitioners' incentives. Practitioners and hospitals can get paid more for this procedure. Although C-sections are necessary in some cases, overuse can increase health risks to the mother and baby and lead to unnecessary medical expenditures.

In contrast, in a study in Norway, researchers found that practitioners there may lack sufficient incentives to be more productive or to provide high-quality services. It mainly manifests in high absenteeism rates among practitioners and nurses in Norway because they do not have sufficient financial incentives to maintain high work engagement and motivation (47). These two examples show the drawbacks of over-reliance on floating parts or fixed parts, and they support the finding of our study: a wage structure that balances the fixed and floating parts can help healthcare organizations avoid over—and under-incentive problems.

**Figure 5:**
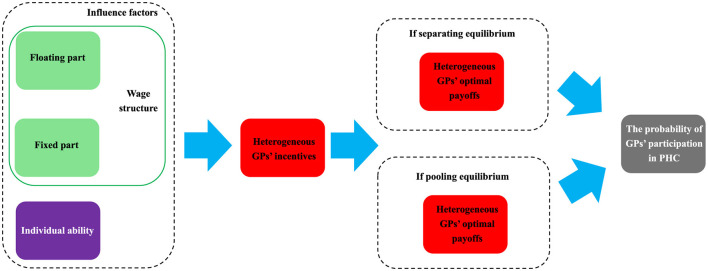
The influence path of wage structure and individual ability on GPs participation in PHC.

Through Proposition 1 and Proposition 2, we can derive the following policy implications. Firstly, after establishing the separation equilibrium mechanism, there are two ways to increase the performance wage of GPs: (1) Narrowing the gap in ability between GPs and high-quality GPs, i.e., by increasing the learning costs of less experienced GPs and raising their abilities; (2) At the same time, improving the performance level of GPs and reducing their performance risk. When the quality of GPs is the same, they are more willing to choose places with lower performance risk. (3) According to the findings of Proposition 1, a transparent promotion system is crucial for GPs to choose to work at PHC institutions. When less experienced GPs increase their abilities by increasing learning costs, they can be promoted from less experienced GPs to high-quality GPs through the incentive mechanism (θ_*L*_ → θ_*H*_). There is a key point worth noting here: we allow θ_*L*_ = θ_*H*_, rather than just letting θ_*L*_ → θ_*H*_. It implies that it is a promotion mechanism. Less experienced GPs can become high-quality GPs by increasing their learning costs. (4) Salary structure reform is necessary. To develop the primary healthcare, it is essential to alter the proportion of fixed and floating parts in the wage structure of grassroots practitioners. Only by reducing the proportion of the fixed part based on the existing wage structure can more high-quality practitioners be attracted. (5) A lower proportion of the floating part is not necessarily better; effort costs influence the optimal wage structure. In other words, the optimal wage structure is influenced by the effective utilization of medical resources. Figure 5 summarizes the influence path of wage structure and individual ability on GPs participation in PHC.

While performance-based incentives play a crucial role in enhancing the efficiency of primary healthcare, excessive reliance on performance metrics can lead to negative consequences.

First, the intrinsic motivation of GPs, including job satisfaction and professional autonomy, may be undermined. When performance evaluations rely too heavily on quantifiable indicators, GPs may shift their focus toward meeting numerical targets at the expense of overall healthcare quality.

Second, excessive emphasis on performance-based incentives may cause GPs to experience stress and burnout. In primary healthcare settings, patient interactions require time and personalized attention. A rigid performance evaluation system may pressure GPs to prioritize short-term outcomes over long-term service quality. Increased performance pressure could further decrease job satisfaction and professional fulfillment.

Third, overemphasis on performance metrics poses a risk of overtreatment. When performance incentives are closely tied to service volume, GPs may be compelled to increase unnecessary medical interventions, such as excessive diagnostic tests, prescriptions, or procedures, to meet predefined performance thresholds. This not only adds financial strain to the healthcare system but may also expose patients to unnecessary costs. Over-reliance on performance metrics can distort clinical decision-making and ultimately reduce healthcare quality.

To mitigate these risks, a balanced incentive structure that integrates both financial and non-financial motivational factors must be adopted. In addition to performance-based compensation, policies should consider career development opportunities, work-life balance, and professional support systems. A hybrid evaluation framework that combines quantitative indicators with qualitative assessments—such as patient feedback, peer reviews, and professional growth indicators—can help improve the balance between quality and performance.

One possible refinement to the incentive system is the incorporation of regional or location-specific contract terms. Given the heterogeneity in healthcare demands across different regions, tailoring contract terms to local conditions could enhance participation in PHC. For instance, relocation bonuses could be offered to incentivize practitioners to work in underdeveloped regions, helping to counteract the “siphon effect” where doctors migrate toward wealthier urban centers. Moreover, performance metrics tailored to regional healthcare needs—such as chronic disease management incentives in rural areas—could improve the alignment between incentives and actual healthcare outcomes.

From an economic perspective, regionalized contracts introduce an additional layer of information asymmetry, where contract efficiency depends on the precise calibration of incentives. If performance-based metrics do not accurately reflect the challenges and resource constraints of different regions, they may lead to unintended distortions in practitioner behavior. Additionally, excessive reliance on location-based incentives might create dependency effects, making it difficult for healthcare institutions to sustain workforce retention in the long term without continuous financial subsidies.

However, when carefully designed, these contracts could mitigate some of the limitations of uniform national-level wage structures. A potential approach would be to combine fixed base wages with region-specific floating incentives, ensuring that practitioners have both income stability and sufficient motivation to adapt to local healthcare priorities. Future studies could explore the optimal balance of these regional contract elements to maximize PHC workforce stability while minimizing inefficiencies.

Based on the above discussion and our simulation results from China, we propose the following potential measures for discussion:

1. Chinese PHC institutions should establish a clear performance-based wage system. Currently, some regions in China employ fee-for-service and fee-for-numbers performance mechanisms, while fixed wage systems dominate most PHC institutions. GPs' quality varies widely. A performance-based wage system with a higher proportion of the floating part under clear separating equilibrium can encourage more GP participation.
2. China's medical education system still needs further improvement, particularly in providing more specialized and long-term training. Presently, training in China is largely short-term and lacks systematic and sustained apprenticeship.
3. Comprehensive reform of wage structures in Chinese medical institutions is necessary. Currently, performance-based pay predominates in hospitals, while fixed-wage pay is common in PHC institutions. In other words, there is excessive fluctuation in hospital physician wages leading to significant wastage of medical resources, while high fixed parts in PHC fail to incentivize GPs. China should reduce the floating part of hospital physician wages (e.g., by implementing annual wages) and lower the fixed part of GP wages (e.g., by adopting performance-based pay). Such reforms in wage structures can attract more doctors to participate in PHC.
4. Finally, managers should mitigate performance risks for less experienced GPs in Chinese PHC institutions. In other words, efforts should focus on improving GP working conditions, clarifying career advancement paths, and enhancing non-financial incentives.

We acknowledge that an alternative approach—offering a higher fixed wage component for underserved areas combined with tailored performance metrics—may also be effective. A higher fixed wage can provide financial stability, reducing uncertainty and making rural placements more attractive, especially for early-career GPs or those less willing to take on performance-based risks.

One potential hybrid approach is the adoption of an annual salary system combined with year-end performance incentives. This model ensures a stable income baseline, reducing financial stress for GPs in underserved areas while maintaining an incentive mechanism that rewards performance over a longer evaluation period. Compared to purely floating wage structures, an annual salary system mitigates short-term income fluctuations, which may be crucial for attracting GPs to rural regions. Meanwhile, year-end performance incentives can be tailored to local healthcare needs, such as chronic disease management or preventive care, ensuring that quality and service delivery remain a priority.

Therefore, the optimal wage structure may depend on regional healthcare conditions and GP preferences. In high-demand urban areas, a higher floating component may be more effective in driving productivity and competition, whereas in underserved rural areas, a balance between an annual salary system and performance-based year-end bonuses may yield better retention and service quality outcomes.

## 4 Conclusion

We analyzed the reasons for the shortage of GPs in China and proposed solutions through a mixed model of adverse selection and moral hazard. Propositions 1 and 2 answered our two research questions. We analyzed the performance mechanism under the separating equilibrium. We could distinguish between performance wage for high-quality and less experienced practitioners by designing a separating equilibrium contract. Proposition 1 revealed that high-quality practitioners experience no distortion at the top, while less experienced practitioners experience distortion in allocation, indicating the existence of second-best contracts. The performance wage of less experienced practitioners is negatively correlated with their quality. When less experienced practitioners increase their ability, their performance wage also increases. Additionally, performance risk is also a crucial factor influencing performance wage. The higher the risk, the lower the performance wage applicable to both types of practitioners.

Furthermore, we found that a suitable promotion mechanism is crucial in influencing practitioners' choices. Through Proposition 2, we proposed a scheme to promote the development of GPs: reforming the wage structure of GPs. We found that reducing the proportion of the fixed part in GPs' wage structure can have positive effects: (1) It can increase the incentive for high-quality practitioners to participate in PHC; (2) It can promote less experienced practitioners to improve their ability. However, the proportion of the fixed part should be reasonable, as it may lead to practitioners increasing the ineffective use of medical resources for performance. The proportion of the fixed part is influenced by effort costs (output costs). We obtained the wage structure under the optimal performance wage. Once PHC institutions adopt the performance mechanism under separating equilibrium, our optimal wage structure can provide a scientific theoretical basis for wage structure reform.

Our innovation lies in laying a theoretical foundation for PHC management, especially the development of GPs, through systematic dynamic analysis. We analyzed the current situation in China facing the development of GPs and proposed solutions through tripartite evolutionary game analysis. We supplemented empirical studies with theoretical foundations. Our findings can provide a scientific basis for future empirical research. Moreover, our theoretical findings can assist government decision-making. The limitation of this study is that first, we only analyzed the ability factors in the motivation of GPs' choices. Other important factors, for example, factors such as the educational resources in the city, convenience of daily life, and whether the GP can live with their family, fairness of performance systems, and the impact of doctor-patient relationships on GP choices, can be explored as essential research topics in the future. We will use empirical data and method (such as qualitative study, discrete choice experiments method to study the impact of non-financial incentives) in subsequent research to verify the correctness of the above theories. Second, we only differentiate between rural and developed areas without considering the nuanced variations across different regions in China. Future research can further study the impact of regional disparities on GP incentive contracts. Based on our theoretical framework, future studies could incorporate empirical data from various regions to optimize GP incentive mechanisms, ensuring a better alignment between compensation structures and local healthcare needs. Future research could also extend our framework to other countries, but due to institutional and labor market differences, the current model should be interpreted as context-specific to China.

## Data Availability

The original contributions presented in the study are included in the article/Supplementary material, further inquiries can be directed to the corresponding author.
