# Supplementary material for: The impact of wage structure and ability on the incentives for practitioners to engage in primary care in China: a mathematical analysis based on the incentive mechanism of heterogeneous practitioners and tripartite evolutionary game
Source: Front Public Health. 2025 Jun 18;13:1527676. doi: 10.3389/fpubh.2025.1527676 (PMC12213744; doi:10.3389/fpubh.2025.1527676)
Supplement: Supplementary file 1 [file Data_Sheet_1.docx]

The first step is to obtain the optimal payoff of GPs under separating equilibrium and pooling equilibrium through the mixed model of adverse selection and moral hazard. In the second step, we analyze whether GPs participate in PHC when PHC institutions adopt the performance mechanism under separating and pooling equilibrium, respectively. In the second step, the heterogeneous GPs’ payoffs come from the optimal payoff that we get in the separating and pooling equilibrium in the first step and then play a tripartite evolutionary game with PHC institutions. Our modeling structure is shown in FIG.1:

**Figure 1 The whole model structure in the study**

**Step 1**, we make assumptions about the contract scenario. According to Jing and Fang [16], the wage structure for China’s GPs consists of three levels. The first level is the medical insurance payment method (external incentive); medical insurance payments are proportionally reimbursed to insurance institutions by patients (second level); then, the medical institutions pay practitioners (third level). Therefore, practitioners establish contractual relationships with medical institutions (It is a difference from our assumption compared to Jing and Fang [16]). In other words, medical institutions commission practitioners to sign contracts to maximize profits. The principal is the medical institution where the GP works, and the agent is the GP. The principal is assumed to be risk-neutral, and its total revenue is a function of the agent's effort $\mathcal{e}$. According to Jing and Fang [16], suppose a GP's effort is $\mathcal{e}\left( t \right)$. We can define the total benefit function for a medical institution as: $Q\left( \mathcal{e}\left( t \right) \right)=\beta\mathcal{e}\left( t \right)+\xi$, where $\beta$ represents the sensitivity of medical institution benefit to the effort exerted by a GP. $\xi$ is the noise term with $0$ mean. a GP also needs to incur effort costs simultaneously. Assume that the effort cost function is $\frac{1}{2}c\mathcal{e}^{2}\left( t \right)$, $c>0$ represents the effort efficiency of a GP. The coefficient encompasses the cost of inefficient medical resource utilization. Due to excessive incentives, efforts to use medical resources inefficiently increase proportionally, leading to wastage. We suppose that the utility function of a GP is CARA form: $u\left( x \right)=-\mathcal{e}^{-\rho P\left( \mathcal{e} \right)}$, $\rho$ represents the absolute risk aversion coefficient. There is a linear relationship between doctor effort and performance as follows: $P\left( \mathcal{e} \right)=B\mathcal{e}\left( t \right)+\zeta$, where $B>0$ is the GP’s performance coefficient, $\zeta$ represents a noise term with a mean of $0$ and a variance of $\sigma^{2}$. Thus, the real wage of a GP is: $W\left( t \right)=mw_{0}+\left( 1-m \right)wP\left( \mathcal{e} \right)$, $w_{0}$ is the basic wage, $m$ represent represents the weight coefficient of the wage structure. Another crucial assumption is that the performance wage of practitioners is correlated with their level of education. This assumption is drawn from Yi et al. [17], where empirical research revealed that factors such as "*uneven allocation of positions within the medical system, a lack of belonging for personnel outside the system, limited professional skills among some family medicine personnel, and uncertain career prospects*" are essential influencers of practitioners' income ([17], p.1228). The agent's net earnings are calculated as the real wage minus effort costs and minus certainty equivalence, namely:

$$\pi_{a}=mw_{0}+\left( 1-m \right)wB\mathcal{e}\left( t \right)-\frac{1}{2}c\mathcal{e}^{2}\left( t \right)-\frac{1}{2}\rho\sigma^{2}w^{2}\left( t \right)$$

Where $\frac{1}{2}\rho w^{2}\left( t \right)$ is the cost of certainty equivalence. In our research question, in addition to involving moral hazard, there is also the issue of adverse selection. We assume that GPs’ ability can be divided into high quality and low quality, that is, the types of practitioners are denoted as $\theta=\left\{ \theta_{H}, \theta_{L} \right\}$. Furthermore, we assume that the output of high-quality GPs is strictly greater than that of low-quality GPs, namely, $\mathcal{e}_{H}>\mathcal{e}_{L}$. Thus, the net payoff of GPs:

$$\pi_{j}=\theta_{j}\left( mw_{0, j}+\left( 1-m \right)w_{j}B_{j}\mathcal{e}_{j}\left( t \right) \right)-\frac{1}{2}c\mathcal{e}_{j}^{2}\left( t \right)-\frac{1}{2}\rho\sigma_{j}^{2}w_{j}^{2}\left( t \right), j\in\left\{ H, L \right\}$$

Therefore, we construct a principal-agent model under incomplete information, and the optimal payoff of the principal in this model will serve as the payoff in the subsequent evolutionary game analysis. The principal's objective function is denoted as $\pi^{SE}$, representing the principal's net profit under the separating equilibrium.

$$\max_{w_{j}} \left\{ \pi^{SE}=P\left[ \beta\mathcal{e}_{H}\left( t \right)-\theta_{H}\left( mw_{0}\left( t \right)+\left( 1-m \right)B_{H}w_{H}\left( t \right)\mathcal{e}_{H}\left( t \right) \right) \right]+\left( 1-P \right)\left[ \beta\mathcal{e}_{L}\left( t \right)-\theta_{L}\left( mw_{0}\left( t \right)+\left( 1-m \right)B_{L}w_{L}\left( t \right)\mathcal{e}_{L}\left( t \right) \right) \right] \right\}$$

$$s.t. IR\left( 1 \right):\pi_{H}^{SB}=\theta_{H}\left( mw_{0, H}+\left( 1-m \right)\left( B_{H}w_{H}-w_{H}^{2}k \right)\mathcal{e}_{H}\left( t \right) \right)-\frac{1}{2}c\mathcal{e}_{H}^{2}\left( t \right)-\frac{1}{2}\rho\sigma_{H}^{2}w_{H}^{2}\left( t \right)\geq0$$

$$IR\left( 2 \right):\pi_{L}^{SB}=\theta_{L}\left( mw_{0, L}+\left( 1-m \right)\left( B_{L}w_{L}-w_{L}^{2}k \right)e_{L}\left( t \right) \right)-\frac{1}{2}c\mathcal{e}_{L}^{2}\left( t \right)-\frac{1}{2}\rho\sigma_{L}^{2}w_{L}^{2}\left( t \right)\geq0$$

$$IC\left( 1 \right):\mathcal{e}_{H}^{*}=\underset{\hat{e}_{H}}{argmax} \left\{ \theta_{H}\left( mw_{0, H}+\left( 1-m \right)\left( B_{H}w_{H}-w_{H}^{2}k \right){\hat{\mathcal{e}}}_{H}\left( t \right) \right)-\frac{1}{2}c{\hat{\mathcal{e}}}_{H}^{2}\left( t \right)-\frac{1}{2}\rho\sigma_{H}^{2}w_{H}^{2}\left( t \right) \right\}$$

$$IC\left( 2 \right): \mathcal{e}_{L}^{*}=\underset{\hat{e}_{L}}{argmax} \left\{ \theta_{L}\left( mw_{0, L}+\left( 1-m \right)\left( B_{L}w_{L}-w_{L}^{2}k \right){\hat{\mathcal{e}}}_{L}\left( t \right) \right)-\frac{1}{2}c{\hat{\mathcal{e}}}_{L}^{2}\left( t \right)-\frac{1}{2}\rho\sigma_{L}^{2}w_{L}^{2}\left( t \right) \right\}$$

$$IC\left( 3 \right): \theta_{H}\left( mw_{0, H}+\left( 1-m \right)\left( B_{H}w_{H}-w_{H}^{2}k \right)\mathcal{e}_{H}\left( t \right) \right)-\frac{1}{2}c\mathcal{e}_{H}^{2}\left( t \right)-\frac{1}{2}\rho\sigma_{H}^{2}w_{H}^{2}\left( t \right)\geq\theta_{H}\left( mw_{0, L}+\left( 1-m \right)\left( B_{L}w_{L}-w_{L}^{2}k \right)\mathcal{e}_{L}\left( t \right) \right)-\frac{1}{2}c\mathcal{e}_{L}^{2}\left( t \right)-\frac{1}{2}\rho\sigma_{L}^{2}w_{L}^{2}\left( t \right)$$

$$IC\left( 4 \right): \theta_{L}\left( mw_{0, L}+\left( 1-m \right)w_{L}B_{L}\mathcal{e}_{L}\left( t \right) \right)-\frac{1}{2}c\mathcal{e}_{L}^{2}\left( t \right)-\frac{1}{2}\rho\left( 1-m \right)^{2}\theta_{L}^{2}\sigma_{L}^{2}w_{L}^{2}\left( t \right)\geq\theta_{L}\left( mw_{0, H}+\left( 1-m \right)\left( B_{H}w_{H}-w_{H}^{2}k \right)\mathcal{e}_{H}\left( t \right) \right)-\frac{1}{2}c\mathcal{e}_{H}^{2}\left( t \right)-\frac{1}{2}\rho\sigma_{H}^{2}w_{H}^{2}\left( t \right)$$

The implicit assumption here is that a GPs’ effort equals output. $IR(1)$ and $IR(2)$ represent the GP's participation constraints, ensuring that the GP will participate in the game. $IC(1)$ to $IC(4)$ are the incentive-compatible constraints for the GP. $IC(1)$ and $IC(2)$ use a truthful revelation mechanism, meaning that the GP and the medical institution will definitely tell the truth under this mechanism. In other words, under the truthful revelation mechanism, we ensure that the GP will exert maximum effort after contract signing and will not shirk [19]. $IC(3)$ and $IC(4)$ ensure that the payoff of high-quality GPs is higher than those of pretending to be high-quality (actually low-quality) GPs, meaning that GPs have no incentive to disguise their types. The objective function is to maximize the payoff of the medical institution. This is illustrated through the following proposition:

**Proposition 1:** Under the incomplete information, the optimal performance wage of a GP is $\left\{ \begin{aligned} w_{H}\left( t \right)=\frac{\theta_{H}\left( 1-m \right)B_{H}\beta}{\theta_{H}^{2}\left( 1-m \right)^{2}B_{H}^{2}+c\rho\sigma_{H}^{2}} \\ w_{L}\left( t \right)=\frac{\left( 1-P \right)\beta\theta_{L}\left( 1-m \right)B_{L}}{\left( 1-P+P\Delta\theta\right)\left[ \theta_{L}^{2}\left( 1-m \right)^{2}B_{L}^{2}+c\rho\sigma_{L}^{2} \right]} \end{aligned} \right.$.

**Proof, See Appendix A.1.** $∎$

Through proposition 1, we can also obtain the optimal effort: $\left\{ \begin{aligned} \mathcal{e}_{H}^{*}=\frac{\theta_{H}^{2}\left( 1-m \right)^{2}B_{H}^{2}\beta}{c\left[ \theta_{H}^{2}\left( 1-m \right)^{2}B_{H}^{2}+c\rho\sigma_{H}^{2} \right]} \\ \mathcal{e}_{L}^{*}=\frac{\left( 1-P \right)\beta\theta_{L}^{2}\left( 1-m \right)^{2}B_{L}^{2}}{c\left( 1-P+P\Delta\theta\right)\left[ \theta_{L}^{2}\left( 1-m \right)^{2}B_{L}^{2}+c\rho\sigma_{L}^{2} \right]} \end{aligned} \right.$. Under complete information, we can have the principal-agent model:

$$\max_{w_{j}} \left\{ \beta\mathcal{e}_{j}\left( t \right)-\theta_{j}\left( mw_{0}\left( t \right)+\left( 1-m \right)B_{j}w_{j}\left( t \right)\mathcal{e}_{j}\left( t \right) \right) \right\}, j\in\{H, L\}$$

$$s.t. IR:\theta_{j}\left( mw_{0, j}\left( t \right)+\left( 1-m \right)B_{j}w_{j}\left( t \right)\mathcal{e}_{j}\left( t \right) \right)-\frac{1}{2}\rho\sigma_{j}^{2}w_{j}^{2}\left( t \right)-\frac{1}{2}c\mathcal{e}_{j}^{2}\left( t \right)\geq0, j\in\{H, L\}$$

$$IC:\mathcal{e}_{j}^{*}=\underset{\hat{e}_{j}}{argmax} \left\{ \theta_{j}\left( mw_{0, j}\left( t \right)+\left( 1-m \right)B_{j}w_{j}\left( t \right){\hat{\mathcal{e}}}_{j}\left( t \right) \right)-\frac{1}{2}\rho\sigma_{j}^{2}w_{j}^{2}\left( t \right)-\frac{1}{2}c{\hat{\mathcal{e}}}_{j}^{2}\left( t \right) \right\}, j\in\{H, L\}$$

Using the same method, we can have the optimal performance wage of a GP: $w_{j}\left( t \right)=\frac{\theta_{j}\left( 1-m \right)B_{j}\beta}{\theta_{j}^{2}\left( 1-m \right)^{2}B_{j}^{2}+c\rho\sigma_{j}^{2}}$,$j\in\{H, L\}$. We can observe that the optimal performance wage for low-quality GPs is different under complete and incomplete information. We denote the performance wage for high-quality practitioners under incomplete information as $w_{H}^{SB}\left( t \right)$ and under complete information as $w_{H}^{FB}\left( t \right)$. We find that $\frac{\partial w_{H}\left( t \right)}{\partial\Delta\theta}<0$, namely, the greater the difference in abilities among GPs, the lower the performance wage for low-quality GPs. When $\theta_{H}=\theta_{L}$, the performance wage for low-quality GPs under complete information is the same as under incomplete information. In other words, any low-quality GP has an incentive to pretend to be a high-quality GP. High-quality GPs have an incentive to promote information transparency. The more transparent the information is, the more the earnings of high-quality GPs can be separated from those of low-quality GPs. It explains why GPs are actively willing to demonstrate their ability to medical institutions during recruitment (or medical institutions would use strict screening criteria such as educational background, the alma mater's reputation, work experience, etc.). Therefore, in the separating equilibrium, there is a distortion in the optimal performance wage for low-quality practitioners, but there is no information rent; high-quality practitioners have information rent but no distortion. This conclusion simultaneously verifies the universality of the phenomenon of top non-distortion. When $\lim_{\sigma_{j}\to\infty} w_{j}\left( t \right)=0$, $j\in\left\{ L, H \right\}$, it indicates that when the randomness of the undertaken production is too high, GPs will have no incentive to engage in such activities due to the risk-averse.

Similarly, the effort level also applies to the above analysis. It is worth noting that the effort level of high-quality GPs under incomplete information is consistent with the effort level under complete information. On the other hand, the effort level of low-quality GPs is negatively correlated with their quality gap. That is, the lower the professional level of low-quality GPs is, the less they want to make an effort. The design of the practitioners' incentive compatibility constraints illustrates that high (low) quality GPs have no incentive to pretend low (high) quality GPs.

$$w_{L}^{FB}\left( t \right)-w_{L}^{SB}\left( t \right)=\frac{\theta_{L}\left( 1-m \right)B_{L}\beta}{\theta_{L}^{2}\left( 1-m \right)^{2}B_{L}^{2}+c\rho\sigma_{L}^{2}}-\frac{\left( 1-P \right)\beta\theta_{L}\left( 1-m \right)B_{L}}{\left( 1-P \right)\theta_{L}^{2}\left( 1-m \right)^{2}B_{L}^{2}+c\left( 1-P \right)\rho\sigma_{L}^{2}+P\Delta\theta\left[ \theta_{L}^{2}\left( 1-m \right)^{2}B_{L}^{2}+c\rho\sigma_{L}^{2} \right]}=\frac{\beta\theta_{L}\left( 1-m \right)B_{L}P\Delta\theta}{\left( 1-P \right)\theta_{L}^{2}\left( 1-m \right)^{2}B_{L}^{2}+c\left( 1-P \right)\rho\sigma_{L}^{2}+P\Delta\theta\left[ \theta_{L}^{2}\left( 1-m \right)^{2}B_{L}^{2}+c\rho\sigma_{L}^{2} \right]}$$

Only $\theta_{H}=\theta_{L}$, we have $w_{L}^{FB}\left( t \right)=w_{L}^{SB}\left( t \right)$*.* The formula indicates that the smaller the $\Delta\theta$ is, the smaller the gap is, between the performance wage of low-quality GPs in the pooling equilibrium and the separating equilibrium. Therefore, raising the ability of low-quality GPs is one effective way to improve their performance wage. When the ability of low-quality GPs is consistent with that of high-quality GPs, their performance wage is similar to under complete information. We have the following corollary:

**Corollary 2:** There exists an optimal wage proportion $m^{*}$, so that can maximize the GPs’ preference wage, if it can be satisfied the conditions $\Delta\theta=\theta_{H}\left( 1-\frac{\sigma_{L}}{\sigma_{H}}\frac{B_{H}}{B_{L}} \right)$ and $c\in\left( 0, \frac{\theta_{H}^{2}B_{H}^{2}}{\rho\sigma_{H}^{2}} \right]\bigcap\left( 0, \frac{\theta_{L}^{2}B_{L}^{2}}{\rho\sigma_{L}^{2}} \right]$.

**Proof. See Appendix A.2.** $\boldsymbol{∎}$

Corollary 2 yields an intriguing finding: based on the situation in China, the performance mechanism we designed under separation equilibrium does not lead to unlimited growth in performance wage. On the contrary, reducing the proportion of the fixed part initially increases performance income but decreases after reaching a maximum point. In other words, an optimal wage structure exists that maximizes performance income. One potential explanation is that as the proportion of the floating part in the wage structure increases, that is, as $m$ decreases, GPs will increase their efforts (output). The increase in output will further increase performance wages. The increase in performance wages will increase the cost of medical resources $c$. In other words, one of the costs of changing the wage structure is the waste of medical resources. Therefore, when the proportion of the floating part in the wage structure increases to a certain extent, the effort cost that practitioners need to pay will exceed the benefits they receive. The performance wages of practitioners will decrease. Through Inference 2, we also discover the driving force behind narrowing the gap in professional levels between low-quality practitioners and high-quality practitioners. Under the conditions of Inference 2, increasing the performance coefficient of high-quality practitioners means that if someone is a high-quality doctor and produces the same output as a low-quality doctor, they can receive more performance income.

Therefore, to maintain the equation $\Delta\theta=\theta_{H}\left( 1-\frac{\sigma_{L}}{\sigma_{H}}\frac{B_{H}}{B_{L}} \right)$, when $B_{H}$ increases, $\Delta\theta$ must decrease. Because $\theta_{H}$ is a fixed parameter, it can only be maintained to have equality on both sides of the equation by increasing $\theta_{L}$. Based on another practical scenario in China: the total wage amount is stipulated within a specific range; we have the following corollary:

**Corollary 3**：There is a reasonable performance wage range $\left\{ \begin{aligned} \overline{W}\in\left[ \frac{\beta^{2}}{4c}, +\infty\right)\bigcap\left[ \frac{\left( 1-P \right)^{2}\beta^{2}}{4c\left( 1-P+P\Delta\theta\right)^{2}}, +\infty\right)\bigcap\left[ 0, \frac{B_{H}^{4}\theta_{H}^{4}\beta^{2}}{c\left( \sigma_{H}^{2}c\rho+1 \right)^{2}} \right] \\ \underline{W}\in\left[ 0, \frac{\beta^{2}}{4c} \right)\bigcap\left[ 0,\frac{\left( 1-P \right)^{2}\beta^{2}}{4c\left( 1-P+P\Delta\theta\right)^{2}} \right)\bigcap\left[ 0, \frac{\theta_{L}^{4}B_{L}^{4}\left( 1-P \right)^{2}\beta^{2}}{c\left( 1-P+P\Delta\theta\right)^{2}\left( \sigma_{L}^{2}c\rho+\theta_{L}^{2}B_{L}^{2} \right)^{2}} \right] \end{aligned} \right.$ so that maximizes performance wage for GPs.

**Proof See Appendix A.3.** $\boldsymbol{∎}$

Corollary 3 represents a deeper understanding of Proposition 1. Based on the practical situation in China, we have derived reasonable upper and lower bounds for the wage structure. We have identified this reasonable range by establishing an interval that includes the optimal performance wages. Corollary 2 and 3 collectively complement the findings drawn from Proposition 1.

**Step 2**. We have obtained payoff matrices for participants in different scenarios by analyzing separating and pooling equilibria. Our further goal is to investigate how to advance the PHC, namely, how to encourage more practitioners to participate in PHC. Next, we will analyze the dynamic process of participant actions using evolutionary game theory. We model based on the actual situation in China. Due to the existing separating equilibrium, it is difficult for low-quality practitioners to disguise themselves as high-quality practitioners to enter hospitals. We establish a tripartite evolutionary game to study how to develop PHC. Due to incomplete information, practitioners find it challenging to make completely rational decisions in the real world. Therefore, based on this fundamental fact, the assumption of bounded rationality in evolutionary game theory becomes our choice. Suppose that a game space $\Theta=\left( I, \left\{ S_{i} \right\}, \left( u_{i}\left( \cdot\right) \right)_{i\in I}, \theta\right)$, where $I=\left\{ Clinics, High-quality practitioners, Low-quality pratitioners \right\}$ represents a players’ set. $S_{i}=\prod_{h\in H} A\left( h \right)$ is a strategy set, $h\in H$ is an information set, $A=\left\{ separating equilibrium, pooling equilibrium, join, reject \right\}$ is an action set. $u_{i}\left( \cdot\right)$ represents payoff set. Table 1 shows the tripartite strategic game.

**Table 1. The payoff matrix of tripartite strategic game**

|  | | **High-quality practitioners** $\boldsymbol{\times}$ **low-quality practitioners** | | | |
| --- | --- | --- | --- | --- | --- |
|  |  | Join, Join | Join, Reject | Reject, Join | Reject, Reject |
| **PHC institutions** | Performance mechanism under separating equilibrium | $\left( \pi_{JJ}^{SE}, \pi_{H}^{SE},\pi_{L}^{SE} \right)$ | $\left( \pi_{JR}^{SE}, \pi_{H}^{SE},-\theta_{L} \right)$ | $\left( \pi_{RJ}^{SE}, 0,\pi_{L}^{SE} \right)$ | $\left( 0, 0,-\theta_{L} \right)$ |
|  | Performance mechanism under Pooling equilibrium | $\left( \pi_{JJ}^{PE}, \pi_{j}^{PE},\pi_{j}^{PE} \right)$ | $\left( \pi_{JR}^{PE}, \pi_{H}^{PE},-\theta_{L} \right)$ | $\left( \pi_{RJ}^{PE}, 0,\pi_{L}^{PE} \right)$ | $\left( 0, 0,-\theta_{L} \right)$ |

**Note:** $\pi_{JJ}^{SE}=P\left[ \beta\mathcal{e}_{H}\left( t \right)-\theta_{H}\left( mw_{0, H}\left( t \right)+\left( 1-m \right)B_{H}w_{H}\left( t \right)\mathcal{e}_{H}\left( t \right) \right) \right]+\left( 1-P \right)\left[ \beta\mathcal{e}_{L}\left( t \right)-\theta_{L}\left( mw_{0, L}\left( t \right)+\left( 1-m \right)B_{L}w_{L}\left( t \right)\mathcal{e}_{L}\left( t \right) \right) \right]$; $\pi_{RJ}^{SE}=\pi_{RJ}^{PE}=\beta\mathcal{e}_{L}\left( t \right)-\theta_{L}\left( mw_{0, L}\left( t \right)+\left( 1-m \right)B_{L}w_{L}\left( t \right)\mathcal{e}_{L}\left( t \right) \right)$; $\pi_{JR}^{PE}=\pi_{JR}^{SE}=\beta\mathcal{e}_{H}\left( t \right)-\theta_{H}\left( mw_{0, H}\left( t \right)+\left( 1-m \right)B_{H}w_{H}\left( t \right)\mathcal{e}_{H}\left( t \right) \right)$; $\pi_{JJ}^{PE}=P\left[ \beta\mathcal{e}_{H}\left( t \right)-\theta_{H}\left( mw_{0, H}\left( t \right)+\left( 1-m \right)B_{H}w_{H}\left( t \right)\mathcal{e}_{H}\left( t \right) \right) \right]+\left( 1-P \right)\left[ \beta\mathcal{e}_{L}\left( t \right)-\theta_{L}\left( mw_{0, H}\left( t \right)+\left( 1-m \right)B_{H}w_{H}\left( t \right)\mathcal{e}_{L}\left( t \right) \right) \right]$, if pooling to high-quality practitioners; $\pi_{JJ}^{PE}=P\left[ \beta\mathcal{e}_{H}\left( t \right)-\theta_{H}\left( mw_{0, L}\left( t \right)+\left( 1-m \right)B_{L}w_{L}\left( t \right)\mathcal{e}_{H}\left( t \right) \right) \right]+\left( 1-P \right)\left[ \beta\mathcal{e}_{L}\left( t \right)-\theta_{L}\left( mw_{0, L}\left( t \right)+\left( 1-m \right)B_{L}w_{L}\left( t \right)\mathcal{e}_{L}\left( t \right) \right) \right]$, if pooling to low-quality practitioners.

Table 1 implies a natural assumption: high-quality practitioners, if they reject the performance mechanism of PHC institutions, have the option to choose hospitals. On the other hand, if low-quality practitioners reject the mechanism of PHC institutions, they can only face unemployment. Therefore, low-quality practitioners will incur education costs $-\theta_{L}$. Assuming the probability of PHC institutions choosing the performance mechanism under separating equilibrium is $x$ and choosing the performance mechanism under pooling equilibrium is $1-x$, and the probability of high-quality practitioners choosing to join is $y$ while choosing to reject is $1-y$, and the probability of low-quality practitioners choosing to join is $z$ while choosing to reject is $1-z$. We first analyze the PHC institutions. The expected payoffs for PHC institutions in implementing the performance mechanisms under separating and pooling equilibrium are as follows:

$$\Pi_{C}^{SE}=yz\pi_{JJ}^{SE}+y\left( 1-z \right)\pi_{JR}^{SE}+\left( 1-y \right)z\pi_{RJ}^{SE}$$

$$\Pi_{C}^{PE}=yz\pi_{JJ}^{PE}+y\left( 1-z \right)\pi_{JR}^{PE}+\left( 1-y \right)z\pi_{RJ}^{PE}=\pi_{JJ}^{PE}\left( y+z-yz \right)$$

The average payoff can be expressed as:

$$\overline{\Pi}_{C}=x\Pi_{C}^{SE}+\left( 1-x \right)\Pi_{C}^{PE}=xyz\pi_{JJ}^{SB}+xy\left( 1-z \right)\pi_{JR}^{SB}+x\left( 1-y \right)z\pi_{RJ}^{SB}+\left( y+z-yz \right)\left( 1-x \right)\pi_{JJ}^{FB}$$

Then, we have a dynamic replicate process:

$$\frac{dx}{dt}=x\left( \Pi_{C}^{SE}-\overline{\Pi}_{C} \right)=x\left( 1-x \right)\left\{ y\left[ z\left( \pi_{JJ}^{SB}-\pi_{JR}^{SB}-\pi_{RJ}^{SB}+\pi_{JJ}^{FB} \right)+\pi_{JR}^{SB}-\pi_{JJ}^{FB} \right]-z\left( \pi_{JJ}^{FB}-\pi_{RJ}^{SB} \right) \right\}$$

Let $F_{C}\left( x, y, z \right)=\frac{dx}{dt}$, we have:

$$\frac{\partial F_{C}\left( x, y, z \right)}{\partial x}=\left( 1-2x \right)\left\{ y\left[ z\left( \pi_{JJ}^{SB}-\pi_{JR}^{SB}-\pi_{RJ}^{SB}+\pi_{JJ}^{FB} \right)+\pi_{JR}^{SB}-\pi_{JJ}^{FB} \right]-z\left( \pi_{JJ}^{FB}-\pi_{RJ}^{SB} \right) \right\}$$

When $y>\frac{z\left( \pi_{JJ}^{PE}-\pi_{RJ}^{SE} \right)}{z\left( \pi_{JJ}^{SE}-\pi_{JR}^{SE}-\pi_{RJ}^{SE}+\pi_{JJ}^{PE} \right)+\pi_{JR}^{SE}-\pi_{JJ}^{PE}}$, we have $\left. \frac{\partial F_{C}\left( x, y, z \right)}{\partial x} \right|_{x=0}>0$, $\left. \frac{\partial F_{C}\left( x, y, z \right)}{\partial x} \right|_{x=1}<0$. Thus, $x=1$ is an ESS; when $y<\frac{z\left( \pi_{JJ}^{PE}-\pi_{RJ}^{SE} \right)}{z\left( \pi_{JJ}^{SE}-\pi_{JR}^{SE}-\pi_{RJ}^{SE}+\pi_{JJ}^{PE} \right)+\pi_{JR}^{SE}-\pi_{JJ}^{PE}}$, we have $\left. \frac{\partial F_{C}\left( x, y, z \right)}{\partial x} \right|_{x=0}<0$, $\left. \frac{\partial F_{C}\left( x, y, z \right)}{\partial x} \right|_{x=1}>0$. Thus, $x=0$ is an ESS.

For high-quality practitioners, the expected payoffs for high-quality practitioners in choosing to implement the Join and Reject mechanisms are as follows:

$$\Pi_{H}^{J}=xz\pi_{H}^{SE}+x\left( 1-z \right)\pi_{H}^{SE}+\left( 1-x \right)z\pi_{j}^{PE}+\left( 1-x \right)\left( 1-z \right)\pi_{H}^{PE}=x\pi_{H}^{SE}+\left( 1-x \right)\left[ z\pi_{j}^{PE}+\left( 1-z \right)\pi_{H}^{PE} \right], j\in\left\{ L, H \right\}$$

$$\Pi_{H}^{R}=0$$

The average payoff can be expressed as:

$$\overline{\Pi}_{H}=yx\pi_{H}^{SE}+y\left( 1-x \right)\left[ z\pi_{j}^{PE}+\left( 1-z \right)\pi_{H}^{PE} \right], j\in\left\{ L, H \right\}$$

Then, we have a dynamic replicate process:

$$\frac{dy}{dt}=y\left( \Pi_{H}^{J}-\overline{\Pi}_{H} \right)=y\left( 1-y \right)\left[ x\left( \pi_{H}^{SE}-\pi_{H}^{PE} \right)+z\left( \pi_{j}^{PE}-\pi_{H}^{PE} \right)\left( 1-x \right)+\pi_{H}^{PE} \right], j\in\left\{ L, H \right\}$$

Let $F_{H}\left( x, y, z \right)=\frac{dy}{dt}$, we have：

$$\frac{\partial F_{H}\left( x, y, z \right)}{\partial y}=\left( 1-2y \right)\left\{ z\left( \pi_{j}^{PE}-\pi_{H}^{PE} \right)+\pi_{H}^{PE}-x\left[ z\left( \pi_{j}^{PE}-\pi_{H}^{PE} \right)-\left( \pi_{H}^{SE}-\pi_{H}^{PE} \right) \right] \right\}, j\in\left\{ L, H \right\}$$

When $x<\frac{z\left( \pi_{j}^{PE}-\pi_{H}^{PE} \right)+\pi_{H}^{PE}}{z\left( \pi_{j}^{PE}-\pi_{H}^{PE} \right)-\left( \pi_{H}^{SE}-\pi_{H}^{PE} \right)}$, $j\in\left\{ L, H \right\}$, we have $\left. \frac{\partial F_{H}\left( x, y, z \right)}{\partial y} \right|_{y=0}>0$, $\left. \frac{\partial F_{H}\left( x, y, z \right)}{\partial y} \right|_{y=1}<0$. Thus, $y=1$ is an ESS; when $x>\frac{z\left( \pi_{j}^{PE}-\pi_{H}^{PE} \right)+\pi_{H}^{PE}}{z\left( \pi_{j}^{PE}-\pi_{H}^{PE} \right)-\left( \pi_{H}^{SE}-\pi_{H}^{PE} \right)}$, $j\in\left\{ L, H \right\}$, we have $\left. \frac{\partial F_{H}\left( x, y, z \right)}{\partial y} \right|_{y=0}<0$, $\left. \frac{\partial F_{H}\left( x, y, z \right)}{\partial y} \right|_{y=1}>0$. Thus, $y=0$ is an ESS.

For low-quality practitioners, the expected payoffs for low-quality practitioners in choosing to implement the Join and Reject mechanisms are as follows:

$$\Pi_{L}^{J}=xy\pi_{L}^{SE}+x\left( 1-y \right)\pi_{L}^{SE}+\left( 1-x \right)y\pi_{j}^{PE}+\left( 1-x \right)\left( 1-y \right)\pi_{L}^{PE}=x\pi_{L}^{SE}+\left( 1-x \right)\left[ y\pi_{j}^{PE}-\left( 1-y \right)\pi_{L}^{PE} \right], j\in\left\{ L, H \right\}$$

$$\Pi_{L}^{R}=-\theta_{L}$$

The average payoff can be expressed as:

$$\overline{\Pi}_{L}=zx\pi_{L}^{SE}+z\left( 1-x \right)\left[ y\pi_{j}^{PE}-\left( 1-y \right)\pi_{L}^{PE} \right]-\left( 1-z \right)\theta_{L}, j\in\left\{ L, H \right\}$$

Then, we have a dynamic replicate process:

$$\frac{dz}{dt}=z\left( \Pi_{L}^{J}-\overline{\Pi}_{L} \right)=z\left( 1-z \right)\left[ x\left( \pi_{L}^{SE}+\pi_{L}^{PE}-y\pi_{j}^{PE}-y\pi_{L}^{PE} \right)+y\left( \pi_{j}^{PE}+\pi_{L}^{PE} \right)-\pi_{L}^{PE}+\theta_{L} \right], j\in\left\{ L, H \right\}$$

Let $F_{L}\left( x, y, z \right)=\frac{dz}{dt}$, we have：

$$\frac{\partial F_{L}\left( x, y, z \right)}{\partial y}=\left( 1-2y \right)\left[ y\left( \pi_{j}^{PE}+\pi_{L}^{PE} \right)-\pi_{L}^{PE}+\theta_{L}-x\left( y\left( \pi_{j}^{PE}+\pi_{L}^{PE} \right)-\pi_{L}^{SE}-\pi_{L}^{PE} \right) \right], j\in\left\{ L, H \right\}$$

When $x<\frac{y\left( \pi_{j}^{PE}+\pi_{L}^{PE} \right)-\pi_{L}^{PE}+\theta_{L}}{y\left( \pi_{j}^{PE}+\pi_{L}^{PE} \right)-\pi_{L}^{SE}-\pi_{L}^{PE}}$, $j\in\left\{ L, H \right\}$, we have $\left. \frac{\partial F_{L}\left( x, y, z \right)}{\partial z} \right|_{z=0}>0$, $\left. \frac{\partial F_{L}\left( x, y, z \right)}{\partial z} \right|_{z=1}<0$. Thus, $z=1$ is an ESS; when $x>\frac{y\left( \pi_{j}^{PE}+\pi_{L}^{PE} \right)-\pi_{L}^{PE}+\theta_{L}}{y\left( \pi_{j}^{PE}+\pi_{L}^{PE} \right)-\pi_{L}^{SE}-\pi_{L}^{PE}}$, $j\in\left\{ L, H \right\}$, we have $\left. \frac{\partial F_{L}\left( x, y, z \right)}{\partial z} \right|_{z=0}<0$, $\left. \frac{\partial F_{L}\left( x, y, z \right)}{\partial z} \right|_{z=1}>0$.. Thus, $z=0$ is an ESS.

Therefore, we obtain 7 potential ESS points: $\left( 1, 1, 1 \right)$，$\left( 1, 1, 0 \right)$，$\left( 1, 0, 1 \right)$，$\left( 0, 0, 0 \right)$，$\left( 0, 1, 1 \right)$，$\left( 0, 1, 0 \right)$ and $\left( 0, 0, 1 \right)$. We build a Jacobin matrix: $J=\left( \begin{matrix} \frac{\partial F_{C}\left( x, y, z \right)}{\partial x} & \frac{\partial F_{C}\left( x, y, z \right)}{\partial y} & \frac{\partial F_{C}\left( x, y, z \right)}{\partial z} \\ \frac{\partial F_{H}\left( x, y, z \right)}{\partial x} & \frac{\partial F_{H}\left( x, y, z \right)}{\partial y} & \frac{\partial F_{H}\left( x, y, z \right)}{\partial z} \\ \frac{\partial F_{L}\left( x, y, z \right)}{\partial x} & \frac{\partial F_{L}\left( x, y, z \right)}{\partial y} & \frac{\partial F_{L}\left( x, y, z \right)}{\partial z} \end{matrix} \right)$. Using Lyapunov method, we have the following proposition:

**Proposition 2:** $\left( 1, 1, 1 \right)$ is an ESS, if it satisfies the condition: $\pi_{JJ}^{PE}<\pi_{JJ}^{SE}$; $\left( 1, 0, 1 \right)$ is an ESS, if it satisfies the condition: $\left\{ \begin{aligned} \pi_{JJ}^{PE}<\pi_{RJ}^{SE} \\ \pi_{H}^{SE}<0 \end{aligned} \right.$; $\left( 0, 0, 1 \right)$ is an ESS, if it satisfies the condition: $\left\{ \begin{aligned} \pi_{RJ}^{SE}<\pi_{JJ}^{PE} \\ \pi_{j}^{SE}<0 \end{aligned} \right.$; $\left( 0, 1, 1 \right)$ is an ESS point, if it satisfies the condition: $\pi_{JJ}^{SE}<\pi_{JJ}^{PE}$.

**Proof. See Appendix A.4.** $\boldsymbol{∎}$

We analyzed, through Proposition 1, the optimal wages obtained by PHC institutions when implementing performance mechanisms under separating equilibrium for different types of practitioners. Proposition 2 provides insights into whether practitioners will choose to remain in PHC institutions when implementing these two different mechanisms. When $w_{0, L}\left( t \right)=w_{0, H}\left( t \right)$, if PHC institutions adopt a strategy of providing high-quality performance wages regardless of the type of practitioner, then we have:

**Corollary 4:** $\left( 1, 1, 1 \right)$ is an ESS, only if $\theta_{L}\in\left[ 0, \frac{1-P}{P} \right)$.

**Proof See Appendix A.5.** $\boldsymbol{∎}$

We find that $(1, 1, 1)$ is an Evolutionarily Stable Strategy (ESS) point. Meanwhile, $(0, 1, 1)$ is not an ESS point. For high-quality practitioners, a "more work, more pay" mechanism incentivizes them to work in PHC institutions. Low-quality practitioners will incur educational costs once they leave PHC institutions. Therefore, they have no incentive to leave PHC institutions regardless. Conversely, if medical institutions adopt a performance wage system where low-quality practitioners receive the same performance wages regardless of their type, in this case, we find that (1,1,1) is not an ESS point. PHC institutions can obtain high-quality output while only paying low-quality wages. PHC institutions will have no incentive to implement a performance wage mechanism under the separating equilibrium. Therefore, in Proposition 2, on the one hand, we find that PHC institutions have an incentive to implement performance mechanisms under separating or pooling equilibriums, especially when high-quality practitioners constitute a more significant proportion. However, when PHC institutions adopt a performance wage system that pays low-quality wages regardless of practitioner type, the net income for high-quality practitioners is $\theta_{H}\left( mw_{0, L}+\left( 1-m \right)w_{L}B_{L}\mathcal{e}_{H}\left( t \right) \right)-\frac{1}{2}c\mathcal{e}_{H}^{2}\left( t \right)-\frac{1}{2}\rho\sigma_{H}^{2}w_{L}^{2}\left( t \right)<0$, namely, $\pi_{j}^{SE}<0$. Thus, under this condition, $(0, 1, 1)$ does not satisfy the Lyapunov stability criterion. $(0, 1, 1)$ is not an ESS. We compare:

$$\pi_{JJ}^{SE}-\pi_{RJ}^{SE}=P\left\{ \left[ \beta\mathcal{e}_{H}\left( t \right)-\theta_{H}\left( mw_{0, H}\left( t \right)+\left( 1-m \right)B_{H}w_{H}\left( t \right)\mathcal{e}_{H}\left( t \right) \right) \right]-\left[ \beta\mathcal{e}_{L}\left( t \right)-\theta_{L}\left( mw_{0, L}\left( t \right)+\left( 1-m \right)B_{L}w_{L}\left( t \right)\mathcal{e}_{L}\left( t \right) \right) \right] \right\}>0\Longrightarrow\pi_{JJ}^{SE}>\pi_{RJ}^{SE}$$

We find that $(1, 1, 1)$ is not an ESS point. PHC institutions can achieve high-quality output while only paying low-quality wages for the high-quality practitioners. The PHC institutions would have no incentive to implement the performance mechanism under separating equilibrium. Therefore, in Proposition 2, on the one hand, we obtain incentives for PHC institutions to implement performance mechanisms under separating or pooling equilibrium. When high-quality practitioners constitute a larger proportion, PHC institutions are more likely to implement performance mechanisms under separating equilibrium. However, when PHC institutions choose to provide low-quality performance wages regardless of the type of practitioner, the net profit for high-quality practitioners is: $\theta_{H}\left( mw_{0, L}+\left( 1-m \right)w_{L}B_{L}\mathcal{e}_{H}\left( t \right) \right)-\frac{1}{2}c\mathcal{e}_{H}^{2}\left( t \right)-\frac{1}{2}\rho\sigma_{H}^{2}w_{L}^{2}\left( t \right)<0$, that is $\pi_{j}^{SE}<0$. Therefore, under the condition, $\left( 0, 1, 1 \right)$ cannot satisfy the judgment of Lyapunov method. $\left( 0, 1, 1 \right)$ is not an ESS. We further compare $\pi_{JJ}^{SE}$ with $\pi_{RJ}^{SE}$:

$$\pi_{JJ}^{SE}-\pi_{RJ}^{SE}=P\left\{ \left[ \beta\mathcal{e}_{H}\left( t \right)-\theta_{H}\left( mw_{0, H}\left( t \right)+\left( 1-m \right)B_{H}w_{H}\left( t \right)\mathcal{e}_{H}\left( t \right) \right) \right]-\left[ \beta\mathcal{e}_{L}\left( t \right)-\theta_{L}\left( mw_{0, L}\left( t \right)+\left( 1-m \right)B_{L}w_{L}\left( t \right)\mathcal{e}_{L}\left( t \right) \right) \right] \right\}>0\Longrightarrow\pi_{JJ}^{SE}>\pi_{RJ}^{SE}$$

It means if satisfied the condition: $\pi_{JJ}^{SE}<\pi_{JJ}^{PE}\Longrightarrow\pi_{RJ}^{SE}<\pi_{JJ}^{PE}$. In this scenario, there is only one unique ESS: $(0, 0, 1)=(1, 0, 1)$. PHC institutions are more likely to implement performance mechanisms under pooling equilibrium when low-quality practitioners constitute a larger proportion. On the other hand, we find that in $(1, 0, 1)$ and $(0, 0, 1)$, the PHC institutions’ payoff $\pi_{RJ}^{SE}$ is equal to the payoff brought by the contract under complete information. In other words, the contracts signed by PHC institutions with low-quality practitioners in $(1, 0, 1)$ and $(0, 0, 1)$ are the same as those signed under complete information. The conditions regarding practitioners in these two points are apparent because practitioners exiting the PHC institutions imply that their participation constraints are strictly less than $0$. Table 2 summarizes possible ESS points. Proposition 2 answers under what conditions PHC institutions can attract more practitioners. For the development of primary healthcare in China, currently, we hope to have more practitioners involved, regardless of their types. Therefore, $(1, 1, 1)$ is the ESS points we aim to achieve.

**Table 2. The points and their ESS judgements**

| **Points** | **ESS** |
| --- | --- |
| $\left( 1, 1, 1 \right)$ | ESS |
| $\left( 1, 0, 1 \right)$ | ESS |
| $\left( 1, 1, 0 \right)$ | / |
| $\left( 1, 0, 0 \right)$ | / |
| $\left( 0, 0, 1 \right)$ | ESS |
| $\left( 0, 1, 0 \right)$ | / |
| $\left( 0, 0, 0 \right)$ | / |
| $\left( 0, 1, 1 \right)$ | / |

**Appendix A**

A.1. proof of proposition 1

$\frac{\partial}{\partial\mathcal{e}_{H}\left( t \right)}=\left( 1-m \right)\theta_{H}B_{H}w_{H}\left( t \right)-c\mathcal{e}_{H}=0\Longrightarrow\mathcal{e}_{H}^{*}=\frac{\left( 1-m \right)\theta_{H}B_{H}w_{H}\left( t \right)}{c}$ (A.1.1)

$\frac{\partial}{\partial\mathcal{e}_{i, L}\left( t \right)}=\theta_{L}B_{L}w_{L}\left( t \right)-c\mathcal{e}_{L}=0\Longrightarrow\mathcal{e}_{L}^{*}=\frac{\left( 1-m \right)\theta_{L}B_{L}w_{L}\left( t \right)}{c}$ (A.1.2)

$\theta_{H}\left( mw_{0, L}\left( t \right)+\left( 1-m \right)B_{L}w_{L}\left( t \right)\mathcal{e}_{L}\left( t \right) \right)-\frac{1}{2}\rho\sigma_{L}^{2}w_{L}^{2}\left( t \right)-\frac{1}{2}c\mathcal{e}_{L}^{2}\left( t \right)=\theta_{H}\left( mw_{0, L}\left( t \right)+\left( 1-m \right)B_{L}w_{L}\left( t \right)\mathcal{e}_{L}\left( t \right) \right)+\theta_{L}\left( mw_{0, L}\left( t \right)+\left( 1-m \right)B_{L}w_{L}\left( t \right)\mathcal{e}_{L}\left( t \right) \right)-\theta_{L}\left( mw_{0, L}\left( t \right)+\left( 1-m \right)B_{L}w_{L}\left( t \right)\mathcal{e}_{L}\left( t \right) \right)-\frac{1}{2}\rho\sigma_{L}^{2}w_{L}^{2}\left( t \right)-\frac{1}{2}c\mathcal{e}_{L}^{2}\left( t \right)=\Delta\theta\left( mw_{0, L}\left( t \right)+\left( 1-m \right)B_{L}w_{L}\left( t \right)\mathcal{e}_{L}\left( t \right) \right)+\pi_{a, L}$ (A.1.3)

$\theta_{L}\left( mw_{0, H}\left( t \right)+\left( 1-m \right)B_{H}w_{H}\left( t \right)\mathcal{e}_{H}\left( t \right) \right)-\frac{1}{2}\rho\sigma_{H}^{2}w_{H}^{2}\left( t \right)-\frac{1}{2}c\mathcal{e}_{H}^{2}\left( t \right)=-\Delta\theta\left( mw_{0, H}\left( t \right)+\left( 1-m \right)B_{H}w_{H}\left( t \right)\mathcal{e}_{H}\left( t \right) \right)+\pi_{a, H}$ (A.1.4)

$\frac{\partial^{2}}{\partial\mathcal{e}_{j}^{2}\left( t \right)}<0$。Thus, there exists a maximum value. We substitute (A.1.1) and (A.1.2) into $IR\left( 1 \right)$ and $IR\left( 2 \right)$:

$$w_{0, j}\left( t \right)=\frac{1}{2\theta_{j}m}\rho\sigma_{j}^{2}w_{j}^{2}\left( t \right)+\frac{1}{2\theta_{j}m}c\mathcal{e}_{j}^{2}\left( t \right)-\frac{1-m}{m}B_{j}w_{j}\left( t \right)\mathcal{e}_{j}\left( t \right), j\in\left\{ H, L \right\}$$

First, we have $\Delta\theta\left( mw_{0, L}\left( t \right)+\left( 1-m \right)B_{L}w_{L}\left( t \right)\mathcal{e}_{L}\left( t \right) \right)+\pi_{a, L}>0$，we can obtain $IR\left( 1 \right)>0$. Thus, $IC\left( 3 \right)$ is an equation constraint. Second, due to $\mathcal{e}_{H}>\mathcal{e}_{L}$, we have $IC\left( 4 \right)>0$, and $IR\left( 2 \right)$ is an equation constraint. Namely $\pi_{a, L}=0$.Therefore, we have:$\pi_{a, H}=\Delta\theta\left( mw_{0, L}\left( t \right)+\left( 1-m \right)B_{L}w_{L}\left( t \right)\mathcal{e}_{L}\left( t \right) \right)\Longrightarrow\frac{1}{2}\rho\sigma_{H}^{2}w_{H}^{2}\left( t \right)+\frac{1}{2}c\mathcal{e}_{H}^{2}\left( t \right)=\Delta\theta\left( mw_{0, L}\left( t \right)+\left( 1-m \right)B_{L}w_{L}\left( t \right)\mathcal{e}_{L}\left( t \right) \right)-\theta_{H}\left( mw_{0, H}\left( t \right)+\left( 1-m \right)B_{H}w_{H}\left( t \right)\mathcal{e}_{H}\left( t \right) \right)$.

We then replace $w_{0,j}\left( t \right)$, $j\in\left\{ H, L \right\}$ into the objective function:

$$\pi_{p}=\max_{w_{j}} \left\{ P\left[ \beta\frac{\left( 1-m \right)\theta_{H}B_{H}w_{H}\left( t \right)}{c}-\frac{1}{2}\rho\sigma_{H}^{2}w_{H}^{2}\left( t \right)-\frac{\left( 1-m \right)^{2}\theta_{H}^{2}B_{H}^{2}}{2c}w_{H}^{2}\left( t \right) \right]+\left( 1-P \right)\left[ \beta\frac{\left( 1-m \right)\theta_{L}B_{L}w_{L}\left( t \right)}{c}-\frac{1}{2}\rho\sigma_{L}^{2}w_{L}^{2}\left( t \right)-\frac{\left( 1-m \right)^{2}\theta_{L}^{2}B_{L}^{2}}{2c}w_{L}^{2}\left( t \right) \right]-P\Delta\theta\left( \frac{1}{2}\rho\sigma_{L}^{2}w_{L}^{2}\left( t \right)+\frac{1}{2}\frac{\theta_{L}^{2}B_{L}^{2}}{c}w_{L}^{2}\left( t \right) \right)-\left( 1-P \right)\pi_{a, L} \right\}, j\in\left\{ H, L \right\}$$

$$\frac{\partial\pi_{p}}{\partial w_{H}\left( t \right)}=\beta\frac{\theta_{H}\left( 1-m \right)B_{H}}{c}-\frac{\theta_{H}^{2}\left( 1-m \right)^{2}B_{H}^{2}}{c}w_{H}-\rho\sigma_{H}^{2}w_{H}=0$$

$$\frac{\partial\pi_{p}}{\partial w_{L}\left( t \right)}=\left( 1-P \right)\beta\frac{\theta_{L}\left( 1-m \right)B_{L}}{c}-\left( 1-P \right)\frac{\theta_{L}^{2}\left( 1-m \right)^{2}B_{L}^{2}}{c}w_{L}-\left( 1-P \right)\rho\sigma_{L}^{2}w_{L}-P\Delta\theta\frac{\theta_{L}^{2}\left( 1-m \right)^{2}B_{L}^{2}}{c}w_{L}-P\Delta\theta\rho\sigma_{L}^{2}w_{L}=0$$

$$\Longrightarrow\left\{ \begin{aligned} w_{H}\left( t \right)=\frac{\theta_{H}\left( 1-m \right)B_{H}\beta}{\theta_{H}^{2}\left( 1-m \right)^{2}B_{H}^{2}+c\rho\sigma_{H}^{2}} \\ w_{L}\left( t \right)=\frac{\left( 1-P \right)\beta\theta_{L}\left( 1-m \right)B_{L}}{\left( 1-P+P\Delta\theta\right)\left[ \theta_{L}^{2}\left( 1-m \right)^{2}B_{L}^{2}+c\rho\sigma_{L}^{2} \right]} \end{aligned} \right.$$

$$∎$$

A.2 Proof of corollary 2

$$\left\{ \begin{aligned} \frac{\partial w_{H}\left( t \right)}{\partial m}=\frac{\theta_{H}^{3}\left( 1-m \right)^{2}B_{H}^{3}\beta-\theta_{H}B_{H}\beta c\rho\sigma_{H}^{2}}{\left( \theta_{H}^{2}\left( 1-m \right)^{2}B_{H}^{2}+c\rho\sigma_{H}^{2} \right)^{2}} \\ \frac{\partial w_{L}\left( t \right)}{\partial m}=\frac{\left( 1-P \right)\theta_{L}B_{L}\beta\left[ \left( 1-P \right)\left( 1-m \right)^{2}B_{L}^{2}\theta_{L}^{2}-\left( 1-P+P\Delta\theta\right)c\rho\sigma_{L}^{2} \right]}{\left\{ \left( 1-P \right)\theta_{L}^{2}\left( 1-m \right)^{2}B_{L}^{2}+c\left( 1-P \right)\rho\sigma_{L}^{2}+P\Delta\theta\left[ \theta_{L}^{2}\left( 1-m \right)^{2}B_{L}^{2}+c\rho\sigma_{L}^{2} \right] \right\}^{2}} \end{aligned} \right.$$

Through the first order condition, we can have:

$$\left\{ \begin{aligned} m_{H}^{*}=1-\frac{\sigma_{H}\sqrt{c\rho}}{\theta_{H}B_{H}} \\ m_{L}^{*}=1-\frac{\sigma_{L}\sqrt{c\rho}}{\theta_{L}B_{L}} \end{aligned} \right.$$

Through the second order condition, we can have: $\left\{ \begin{aligned} \frac{\partial^{2}w_{H}\left( t \right)}{\partial m^{2}}<0 \\ \frac{\partial^{2}w_{L}\left( t \right)}{\partial m^{2}}<0 \end{aligned} \right.$. Let $m_{H}^{*}=m_{L}^{*}$, we can obtain: $\theta_{L}B_{L}\sigma_{H}=\theta_{H}B_{H}\sigma_{L}\Longrightarrow\Delta\theta=\theta_{H}\left( 1-\frac{\sigma_{L}}{\sigma_{H}}\frac{B_{H}}{B_{L}} \right)$ and $c\in\left( 0, \frac{\theta_{H}^{2}B_{H}^{2}}{\rho\sigma_{H}^{2}} \right]\bigcap\left( 0, \frac{\theta_{L}^{2}B_{L}^{2}}{\rho\sigma_{L}^{2}} \right]$.

$$∎$$

A.3 Proof of corollary 3

$$\left\{ \begin{aligned} \overline{W}\geq\frac{\theta_{H}^{4}\left( 1-m \right)^{4}B_{H}^{4}\beta^{2}}{{c\left[ \theta_{H}^{2}\left( 1-m \right)^{2}B_{H}^{2}+c\rho\sigma_{H}^{2} \right]}^{2}}\geq\underline{W} \\ \overline{W}\geq\frac{\left( 1-P \right)^{2}\beta^{2}\theta_{L}^{4}\left( 1-m \right)^{4}B_{L}^{4}}{c\left( 1-P+P\Delta\theta\right)^{2}\left[ \theta_{L}^{2}\left( 1-m \right)^{2}B_{L}^{2}+c\rho\sigma_{L}^{2} \right]^{2}}\geq\underline{W} \end{aligned} \right.\Longrightarrow\Longrightarrow\left\{ \begin{aligned} 1-\frac{\sigma_{H}\sqrt{\sqrt{\underline{W}c}c\rho}}{B_{H}\theta_{H}\sqrt{\beta-\sqrt{\underline{W}c}}}\geq m_{H}\geq1-\frac{\sigma_{H}\sqrt{\sqrt{\overline{W}c}c\rho}}{B_{H}\theta_{H}\sqrt{\beta-\sqrt{\overline{W}c}}} \\ 1-\frac{\sigma_{L}\sqrt{\sqrt{\underline{W}c}\left( 1-P+P\Delta\theta\right)c\rho}}{\theta_{L}B_{L}\sqrt{\left( 1-P \right)\beta-\sqrt{\underline{W}c}\left( 1-P+P\Delta\theta\right)}}\geq m_{L}\geq1-\frac{\sigma_{L}\sqrt{\sqrt{\overline{W}c}\left( 1-P+P\Delta\theta\right)c\rho}}{\theta_{L}B_{L}\sqrt{\left( 1-P \right)\beta-\sqrt{\overline{W}c}\left( 1-P+P\Delta\theta\right)}} \end{aligned} \right.$$

Replace the $m_{H}^{*}$ and $m_{L}^{*}$ into the above inequation, we have:

$$\left\{ \begin{aligned} \overline{W}\in\left[ \frac{\beta^{2}}{4c}, +\infty\right)\bigcap\left[ \frac{\left( 1-P \right)^{2}\beta^{2}}{4c\left( 1-P+P\Delta\theta\right)^{2}}, +\infty\right)\bigcap\left[ 0, \frac{B_{H}^{4}\theta_{H}^{4}\beta^{2}}{c\left( \sigma_{H}^{2}c\rho+1 \right)^{2}} \right] \\ \underline{W}\in\left[ 0, \frac{\beta^{2}}{4c} \right)\bigcap\left[ 0,\frac{\left( 1-P \right)^{2}\beta^{2}}{4c\left( 1-P+P\Delta\theta\right)^{2}} \right)\bigcap\left[ 0, \frac{\theta_{L}^{4}B_{L}^{4}\left( 1-P \right)^{2}\beta^{2}}{c\left( 1-P+P\Delta\theta\right)^{2}\left( \sigma_{L}^{2}c\rho+\theta_{L}^{2}B_{L}^{2} \right)^{2}} \right] \end{aligned} \right.$$

$$∎$$

A.4 Proof of proposition 2

Situation 1. Under the point $\left( 1, 1, 1 \right)$, we have the Jacobin matrix: $J=\left( \begin{matrix} \pi_{JJ}^{PE}-\pi_{JJ}^{SE} & 0 & 0 \\ 0 & -\pi_{H}^{SE} & 0 \\ 0 & 0 & -\left( \theta_{L}+\pi_{L}^{SE} \right) \end{matrix} \right)$. According to Lyapunov method, Only the traces are less than $0$, $\left( 1, 1, 1 \right)$ can be an ESS point. Thus, in its Jacobin matrix, we can find when $\pi_{JJ}^{FB}<\pi_{JJ}^{SB}$ satisfied, $\left( 1, 1, 1 \right)$ is an ESS point.

Situation 2. Under the point $\left( 1, 1, 0 \right)$, we have the Jacobin matrix: $J=\left( \begin{matrix} \pi_{JJ}^{PE}-\pi_{JJ}^{SE} & 0 & 0 \\ 0 & -\pi_{H}^{SE} & 0 \\ 0 & 0 & \pi_{L}^{SE}+\theta_{L} \end{matrix} \right)$. If the point $\left( 1, 1, 0 \right)$ is an ESS, the conditions need to be satisfied: $\left\{ \begin{aligned} \pi_{JJ}^{PE}<\pi_{JJ}^{SE} \\ \pi_{L}^{SE}<-\theta_{L} \end{aligned} \right.$. Thus, it is impossible that $\left( 1, 1, 0 \right)$ is an ESS.

Situation 3. Under the point $\left( 1, 0, 1 \right)$, we have the Jacobin matrix: $J=\left( \begin{matrix} \pi_{JJ}^{PE}-\pi_{RJ}^{SE} & 0 & 0 \\ 0 & \pi_{H}^{SE} & 0 \\ 0 & 0 & -\left( w_{L}^{SE}+\theta_{L} \right) \end{matrix} \right)$. If the point $\left( 1, 0, 1 \right)$ is an ESS, the conditions need to be satisfied: $\left\{ \begin{aligned} \pi_{JJ}^{PE}<\pi_{RJ}^{SE} \\ \pi_{H}^{SE}<0 \end{aligned} \right.$.

Situation 4. Under the point $\left( 1, 0, 0 \right)$, we have the Jacobin matrix: $J=\left( \begin{matrix} 0 & 0 & 0 \\ 0 & \pi_{H}^{SE} & 0 \\ 0 & 0 & \pi_{L}^{SE}+\theta_{L} \end{matrix} \right)$. One of its traces is equal to $0$. Thus, it is impossible that $\left( 1, 0, 0 \right)$ is an ESS.

Situation 5. Under the point $\left( 0, 0, 0 \right)$, we have the Jacobin matrix: $J=\left( \begin{matrix} 0 & 0 & 0 \\ 0 & \pi_{L}^{PE} & 0 \\ 0 & 0 & \pi_{L}^{PE}+\theta_{L} \end{matrix} \right)$. One of its traces is equal to $0$. Thus, it is impossible that $\left( 0, 0, 0 \right)$ is an ESS.

Situation 6. Under the point $\left( 0, 0, 1 \right)$, we have the Jacobin matrix: $J=\left( \begin{matrix} \pi_{RJ}^{SE}-\pi_{JJ}^{PE} & 0 & 0 \\ 0 & \pi_{j}^{PE} & 0 \\ 0 & 0 & -\left( \pi_{L}^{PE}+\theta_{L} \right) \end{matrix} \right)$. If the point $\left( 0, 0, 1 \right)$ is an ESS, the conditions need to be satisfied: $\left\{ \begin{aligned} \pi_{RJ}^{SE}<\pi_{JJ}^{PE} \\ \pi_{j}^{PE}<0 \end{aligned} \right.$.

Situation 7. Under the point $\left( 0, 1, 0 \right)$, we have the Jacobin matrix: $J=\left( \begin{matrix} \pi_{JR}^{SE}-\pi_{JJ}^{PE} & 0 & 0 \\ 0 & -\pi_{j}^{PE} & 0 \\ 0 & 0 & \pi_{L}^{PE}+\theta_{L} \end{matrix} \right)$. If the point $\left( 0, 0, 1 \right)$ is an ESS, the conditions need to be satisfied: $\left\{ \begin{aligned} \pi_{JR}^{SE}<\pi_{JJ}^{PE} \\ \pi_{L}^{PE}<-\theta_{L} \end{aligned} \right.$. Thus, it is impossible that $\left( 0, 1, 0 \right)$ is an ESS.

Situation 7. Under the point $\left( 0, 1, 1 \right)$, we have the Jacobin matrix: $J=\left( \begin{matrix} \pi_{JJ}^{SE}-\pi_{JJ}^{PE} & 0 & 0 \\ 0 & -\pi_{j}^{PE} & 0 \\ 0 & 0 & -\left( \pi_{L}^{PE}+\theta_{L} \right) \end{matrix} \right)$. According to Lyapunov method, Only the traces are less than $0$, $\left( 0, 1, 1 \right)$ can be an ESS point. Thus, in its Jacobin matrix, we can find when $\pi_{JJ}^{SE}<\pi_{JJ}^{PE}$ satisfied, $\left( 0, 1, 1 \right)$ is an ESS point.

$$∎$$

A.5 proof of corollary 4

If the institution chooses to pooling to the high-quality practitioners

$$\pi_{JJ}^{SE}-\pi_{JJ}^{PE}=\left( 1-m \right)\left( 1-P \right)\theta_{L}\left[ B_{H}w_{H}^{PE}\left( t \right)-B_{L}w_{L}^{SE}\left( t \right) \right]\mathcal{e}_{L}\left( t \right)\Longrightarrow B_{H}w_{H}^{PE}\left( t \right)-B_{L}w_{L}^{SE}\left( t \right)=\frac{\theta_{H}\left( 1-m \right)B_{H}^{2}\beta}{\theta_{H}^{2}\left( 1-m \right)^{2}B_{H}^{2}+c\rho\sigma_{H}^{2}}-\frac{\left( 1-P \right)\beta\theta_{L}\left( 1-m \right)B_{L}^{2}}{\left( 1-P+P\Delta\theta\right)\left[ \theta_{L}^{2}\left( 1-m \right)^{2}B_{L}^{2}+c\rho\sigma_{L}^{2} \right]}=c\rho\left\{ \left[ \theta_{L}\left( \sigma_{H}B_{L}-B_{H}\sigma_{L} \right)\left( \sigma_{H}B_{L}+B_{H}\sigma_{L} \right)-\Delta\theta B_{H}^{2}\sigma_{L}^{2} \right]\left( 1-P \right)-\theta_{H}B_{H}^{2}P\Delta\theta\sigma_{L}^{2} \right\}-B_{L}^{2}B_{H}^{2}\Delta\theta\theta_{L}\theta_{H}\left( \theta_{L}P-1+P \right)\left( 1-m \right)^{2}$$

If we want to gain $\pi_{JJ}^{SE}>\pi_{JJ}^{PE}$, we need to have:

$$\frac{c\rho\left\{ \left[ \theta_{L}\left( \sigma_{H}B_{L}-B_{H}\sigma_{L} \right)\left( \sigma_{H}B_{L}+B_{H}\sigma_{L} \right)-\Delta\theta B_{H}^{2}\sigma_{L}^{2} \right]\left( 1-P \right)-\theta_{H}B_{H}^{2}P\Delta\theta\sigma_{L}^{2} \right\}}{B_{L}^{2}B_{H}^{2}\Delta\theta\theta_{L}\theta_{H}\left( \theta_{L}P-1+P \right)}>\left( 1-m \right)^{2}\Longrightarrow m>1-\frac{1}{B_{L}B_{H}}\sqrt{\frac{c\rho\left\{ \left[ \theta_{L}\left( \sigma_{H}^{2}B_{L}^{2}-B_{H}^{2}\sigma_{L}^{2} \right)-\Delta\theta B_{H}^{2}\sigma_{L}^{2} \right]\left( 1-P \right)-\theta_{H}B_{H}^{2}P\Delta\theta\sigma_{L}^{2} \right\}}{\Delta\theta\theta_{L}\theta_{H}\left( \theta_{L}P-1+P \right)}}$$

Since we need guarantee $m\in\left[ 0, 1 \right]$, we have $\Delta\theta\in\left( \frac{c\rho\theta_{L}\left( \sigma_{H}^{2}B_{L}^{2}-B_{H}^{2}\sigma_{L}^{2} \right)\left( 1-P \right)}{B_{H}^{2}\left[ B_{L}^{2}\theta_{L}\theta_{H}\left( \theta_{L}P-1+P \right)+c\rho\sigma_{L}^{2}\left( P\theta_{H}+1-P \right) \right]}, \frac{\theta_{L}\left( \sigma_{H}^{2}B_{L}^{2}-B_{H}^{2}\sigma_{L}^{2} \right)\left( 1-P \right)}{\sigma_{L}^{2}B_{H}^{2}\left( 1-P+P\theta_{H} \right)} \right)$. Thus, in general, when $m\in\left( 1-\frac{1}{B_{L}B_{H}}\sqrt{\frac{c\rho\left\{ \left[ \theta_{L}\left( \sigma_{H}^{2}B_{L}^{2}-B_{H}^{2}\sigma_{L}^{2} \right)-\Delta\theta B_{H}^{2}\sigma_{L}^{2} \right]\left( 1-P \right)-\theta_{H}B_{H}^{2}P\Delta\theta\sigma_{L}^{2} \right\}}{\Delta\theta\theta_{L}\theta_{H}\left( \theta_{L}P-1+P \right)}}, 1 \right]$, we can have $\pi_{JJ}^{SE}>\pi_{JJ}^{PE}$, if it satisfied the conditions: $\Delta\theta\in\left( \frac{c\rho\theta_{L}\left( \sigma_{H}^{2}B_{L}^{2}-B_{H}^{2}\sigma_{L}^{2} \right)\left( 1-P \right)}{B_{H}^{2}\left[ B_{L}^{2}\theta_{L}\theta_{H}\left( \theta_{L}P-1+P \right)+c\rho\sigma_{L}^{2}\left( P\theta_{H}+1-P \right) \right]}, \frac{\theta_{L}\left( \sigma_{H}^{2}B_{L}^{2}-B_{H}^{2}\sigma_{L}^{2} \right)\left( 1-P \right)}{\sigma_{L}^{2}B_{H}^{2}\left( 1-P+P\theta_{H} \right)} \right)$. Specifically, following the condition of corollary 2, we have: $\frac{c\rho\sigma_{L}^{2}+B_{L}^{2}\theta_{L}^{2}\left( 1-m \right)^{2}}{\theta_{L}}\Delta\theta B_{H}^{2}\left( 1-P-\theta_{L}P \right)\theta_{H}$, only if $\frac{1-P}{P}>\theta_{L}\geq0$.

If the institution chooses to pooling to the less experienced practitioners

$$\pi_{JJ}^{SE}-\pi_{JJ}^{PE}=P\theta_{H}\left( 1-m \right)\left[ B_{H}w_{H}^{SE}\left( t \right)-B_{L}w_{L}^{PE}\left( t \right) \right]\mathcal{e}_{H}\left( t \right)\Longrightarrow B_{H}w_{H}^{SE}\left( t \right)-B_{L}w_{L}^{PE}\left( t \right)=\frac{\theta_{H}\left( 1-m \right)B_{H}^{2}\beta}{\theta_{H}^{2}\left( 1-m \right)^{2}B_{H}^{2}+c\rho\sigma_{H}^{2}}-\frac{\theta_{L}\left( 1-m \right)B_{L}^{2}\beta}{\theta_{L}^{2}\left( 1-m \right)^{2}B_{L}^{2}+c\rho\sigma_{L}^{2}}=\left( 1-m \right)\beta\frac{-\Delta\theta\left( 1-m \right)^{2}B_{L}^{2}B_{H}^{2}\theta_{H}\theta_{L}+c\rho\left( \theta_{H}B_{H}^{2}\sigma_{L}^{2}-\theta_{L}B_{L}^{2}\sigma_{H}^{2} \right)}{\left[ \theta_{H}^{2}\left( 1-m \right)^{2}B_{H}^{2}+c\rho\sigma_{H}^{2} \right]\left[ \theta_{L}^{2}\left( 1-m \right)^{2}B_{L}^{2}+c\rho\sigma_{L}^{2} \right]}$$

If we want to gain $\pi_{JJ}^{SE}>\pi_{JJ}^{PE}$, we need to have:

$$-\Delta\theta\left( 1-m \right)^{2}B_{L}^{2}B_{H}^{2}\theta_{H}\theta_{L}+c\rho\left( \theta_{H}B_{H}^{2}\sigma_{L}^{2}-\theta_{L}B_{L}^{2}\sigma_{H}^{2} \right)>0\Longrightarrow m>1-\frac{1}{B_{L}B_{H}}\sqrt{\frac{c\rho\left( \theta_{H}B_{H}^{2}\sigma_{L}^{2}-\theta_{L}B_{L}^{2}\sigma_{H}^{2} \right)}{\Delta\theta\theta_{H}\theta_{L}}}$$

Since we need guarantee $m\in\left[ 0, 1 \right]$, we have $\Delta\theta\in\left( \frac{c\rho\left( \theta_{H}B_{H}^{2}\sigma_{L}^{2}-\theta_{L}B_{L}^{2}\sigma_{H}^{2} \right)}{B_{L}^{2}B_{H}^{2}\theta_{H}\theta_{L}}, +\infty\right)$ and $\theta_{H}B_{H}^{2}\sigma_{L}^{2}>\theta_{L}B_{L}^{2}\sigma_{H}^{2}$. Thus, in general, when $m\in\left( 1-\frac{1}{B_{L}B_{H}}\sqrt{\frac{c\rho\left( \theta_{H}B_{H}^{2}\sigma_{L}^{2}-\theta_{L}B_{L}^{2}\sigma_{H}^{2} \right)}{\Delta\theta\theta_{H}\theta_{L}}}, 1 \right]$, we can have $\pi_{JJ}^{SE}>\pi_{JJ}^{PE}$, if it satisfied the conditions: $\pi_{JJ}^{SE}>\pi_{JJ}^{PE}$, if it satisfied the conditions: $\Delta\theta\in\left( \frac{c\rho\left( \theta_{H}B_{H}^{2}\sigma_{L}^{2}-\theta_{L}B_{L}^{2}\sigma_{H}^{2} \right)}{B_{L}^{2}B_{H}^{2}\theta_{H}\theta_{L}}, +\infty\right)$. Specifically, following the condition of corollary 2, we have: $-\left( 1-m \right)\beta\frac{\left( 1-m \right)^{2}B_{H}^{2}\theta_{H}^{2}+c\rho\sigma_{H}^{2}}{\theta_{H}\left[ \theta_{H}^{2}\left( 1-m \right)^{2}B_{H}^{2}+c\rho\sigma_{H}^{2} \right]\left[ \theta_{L}^{2}\left( 1-m \right)^{2}B_{L}^{2}+c\rho\sigma_{L}^{2} \right]}\Delta\theta\theta_{L}B_{L}^{2}<0$. It indicates that there is no probability that the institution would choose the optimal wage structure under the situation.

$$\boldsymbol{∎}$$
